# Characterisation of neurons derived from a cortical human neural stem cell line CTX0E16

**DOI:** 10.1186/s13287-015-0136-8

**Published:** 2015-08-22

**Authors:** Greg W. Anderson, P. J. Michael Deans, Ruth D T Taylor, Pooja Raval, Ding Chen, Harrison Lowder, Srishti Murkerji, Laura C. Andreae, Brenda P. Williams, Deepak P. Srivastava

**Affiliations:** Department of Basic and Clinical Neuroscience, Institute of Psychiatry Psychology & Neuroscience, King’s College London, London, SE5 8AF UK; MRC Centre for Developmental Neurobiology, King’s College London, London, SE5 8AF UK

## Abstract

**Introduction:**

Conditionally immortalised human neural progenitor cells (hNPCs) represent a robust source of native neural cells to investigate physiological mechanisms in both health and disease. However, in order to recognise the utility of such cells, it is critical to determine whether they retain characteristics of their tissue of origin and generate appropriate neural cell types upon differentiation. To this end, we have characterised the conditionally immortalised, cortically-derived, human NPC line, CTX0E16, investigating the molecular and cellular phenotype of differentiated neurons to determine whether they possess characteristics of cortical glutamatergic neurons.

**Methods:**

Differentiated CTX0E16 cells were characterised by assessing expression of several neural fates markers, and examination of developing neuronal morphology. Expression of neurotransmitter receptors, signalling proteins and related proteins were assessed by q- and RT-PCR and complemented by Ca^2+^ imaging, electrophysiology and assessment of ERK signalling in response to neurotransmitter ligand application. Finally, differentiated neurons were assessed for their ability to form putative synapses and to respond to activity-dependent stimulation.

**Results:**

Differentiation of CTX0E16 hNPCs predominately resulted in the generation of neurons expressing markers of cortical and glutamatergic (excitatory) fate, and with a typical polarized neuronal morphology. Gene expression analysis confirmed an upregulation in the expression of cortical, glutamatergic and signalling proteins following differentiation. CTX0E16 neurons demonstrated Ca^2+^ and ERK1/2 responses following exogenous neurotransmitter application, and after 6 weeks displayed spontaneous Ca^2+^ transients and electrophysiological properties consistent with that of immature neurons. Differentiated CTX0E16 neurons also expressed a range of pre- and post-synaptic proteins that co-localized along distal dendrites, and moreover, displayed structural plasticity in response to modulation of neuronal activity.

**Conclusions:**

Taken together, these findings demonstrate that the CTX0E16 hNPC line is a robust source of cortical neurons, which display functional properties consistent with a glutamatergic phenotype. Thus CTX0E16 neurons can be used to study cortical cell function, and furthermore, as these neurons express a range of disease-associated genes, they represent an ideal platform with which to investigate neurodevelopmental mechanisms in native human cells in health and disease.

**Electronic supplementary material:**

The online version of this article (doi:10.1186/s13287-015-0136-8) contains supplementary material, which is available to authorized users.

## Introduction

In the past decade, advances in stem cell biology have led to the emergence of novel powerful tools to investigate complex questions in neurobiology. Human neural stem cells (hNSCs) and the neural progenitor cells (NPCs) that they generate have become a major focus of interest as they provide a renewable and accessible model system in which to investigate basic human neurodevelopment mechanisms and complex neurodevelopmental disorders [1–4]. One major advantage of hNPCs is that they can easily undergo biochemical, pharmacological and genetic manipulations, making them an ideal platform for high-throughput, genetic or small molecule functional screening [1–3, 5, 6].

Human NSCs and NPCs have been derived from numerous stem cell types, including embryonic, fetal and adult stem cells [7–10]. Previous studies have demonstrated that hNPCs are self-renewing and are multipotent, being able to differentiate into multiple neural cell types, including different types of neurons, astroctyes and oligodendrocytes [11–14]. Many groups have successfully generated neurons characteristic of different neural tissues, including spinal motor neurons, spinal cord interneurons, midbrain dopaminergic and cortical pyramidal neurons, from rodent and human embryonic stem cells [11–17]. However, the ethical and logistical considerations associated with the use of human blastocytes, from which embryonic stem cells are derived, often makes this approach difficult, especially when investigating the basic mechanisms underlying neurodevelopment. An alternative approach has been the creation of conditionally immortalised hNPCs, generated from post-mortem human fetal tissue [2, 5, 6, 14]. Recently several clonal, conditionally immortalised hNPC lines were isolated from first trimester human fetal tissues [14]. These cells were conditionally immortalised using retroviral integration of a single copy of the c-mycER^TAM^ construct. Thus, in the presence of 4-hydroxytamoxifen (4-OHT) and defined growth factors, these hNPCs retain their self-renewing properties. However, upon withdrawal of 4-OHT and trophic support, and the addition of a medium that promotes neuronal differentiation, these cells terminally differentiate into functional neurons that retain regional identity [14]. Indeed, immortalised hNPCs isolated from first trimester human fetal spinal cord, midbrain, hippocampus and cortex have been successfully differentiated into functional neurons and interneurons both *in vitro* and *in vivo* [12, 14, 18, 19]. These hNPC lines have already been used to investigate the mechanisms of antidepressant drug action [18], to characterise the biological functions of susceptibility genes for schizophrenia and bipolar disorder [20] and are currently in trial for engraftment following ischaemic stroke [21]. However, in order to fully understand how such mechanisms may impact early neurodevelopment, and in particular corticogenesis, it is critical to determine whether these cell lines can a) robustly differentiate into glutamatergic neurons *in vitro*; b) display functional responses to activation of neurotransmitter signalling systems; c) form putative synapses; and d) are able to respond to activity-dependent stimulations in the anticipated manner.

Here we report that the conditionally immortalised CTX0E16 hNPCs are capable of generating glutamatergic neurons under defined culturing conditions. These neurons expressed a range of neuronal proteins and demonstrated spontaneous and evoked Ca^2+^ responses and activation of intracellular signalling cascades in response to exogenous neurotransmitter application. Critically, differentiated neurons developed electrophysiological properties typical of young, immature neurons. CTX0E16 neurons also demonstrated morphological maturation over time, expressed a range of pre- and post-synaptic proteins, and displayed structural plasticity in response to modulation of neuronal activity. Collectively, these data demonstrate that the CTX0E16 hNPC line is an ideal platform from which investigations into basic and disease-relevant neurodevelopmental mechanisms can be investigated in native human neurons.

## Materials and methods

### Cell culture

Use of the CTX0E16 cell line was kindly granted by ReNeuron Group plc (Guildford, UK) under a Material Transfer Agreement. Derivation of this conditionally immortalised human NPC line has been described previously [14, 19]. In brief, CTX0E16 cells were obtained from the developing embryonic cortex of a 12-week gestation fetus and conditionally immortalised by ectopic expression of the *c*-MycER^TAM^ transgene [14]. This construct maintains self-renewal of the cells in the presence of 4-OHT.

CTX0E16 hNPCs were cultured in reduced modified medium (RMM: DMEM:F12 with 15 mM HEPES and sodium bicarbonate (Sigma, St. Louis, MO, USA) supplemented with 0.03 % human serum albumin (GE Healthcare Life Sciences, Buckinghamshire, UK), 100 μg/ml apo-transferrin (Scipac Ltd, Kent, UK), 16.2 μg/ml putrescine (Sigma, St. Louis, MO, USA), 5 μg/ml human insulin (Sigma, St. Louis, MO), 60 ng/ml progesterone (Sigma, St. Louis, MO, USA), 2 mM L-glutamine (Sigma, St. Louis, MO, USA) and 40 ng/ml sodium selenite (Sigma, St. Louis, MO, USA)). To maintain proliferation 10 ng/ml human fibroblast growth factor (FGF)_2_ (PeproTech, Rocky Hill, NJ), 20 ng/ml human epidermal growth factor (EGF) (PeproTech, Rocky Hill, NJ) and 100 nM 4-OHT (Sigma, St. Louis, MO, USA were also added to RMM. CTX0E16 hNPCs were seeded onto Poly-D-lysine (PDL, 5 μg/cm^2^; Sigma, St. Louis, MO, USA) and laminin-coated (1 μg/cm^2^; Sigma, St. Louis, MO, USA) tissue culture flasks, with full media changes occurring every 2–3 days. Cells were passaged once they reached 70–80 % confluence using Accutase (Sigma, St. Louis, MO, USA) and maintained for between 25 and 30 passages; all experiments were carried out using cells from passages 12 to 30.

### Neural progenitor cell differentiation

CTX0E16 cultures were maintained under proliferative conditions until 70–90 % confluent, then washed twice with non-supplemented DMEM:F12 medium and passaged onto PDL (5 μg/cm^2^) and laminin-coated (1 μg/cm^2^) tissue culture flasks or No. 1.5 coverglass (Paul Marienfeld GmbH & Co. KG, Lauda-Königshofen, Germany) at a density of 50,000 cells per ml. Cells were then washed in warm Dulbecco’s phosphate-buffered saline (DPBS; Life Technologies, Paisley, UK) and maintained in neuronal differentiation media (NDM: Neurobasal Medium (Life Technologies, Paisley, UK) supplemented with 0.03 % human serum albumin (GE Healthcare Life Sciences, Buckinghamshire, UK), 100 μg/ml apo-transferrin (Scipac Ltd, Kent, UK), 16.2 μg/ml putrescine (Sigma, St. Louis, MO, USA), 5 μg/ml human insulin (Sigma, St. Louis, MO, USA), 60 ng/ml progesterone (Sigma, St. Louis, MO, USA), 2 mM L-glutamine (Sigma, St. Louis, MO, USA), 40 ng/ml sodium selenite (Sigma, St. Louis, MO, USA) and 1 × B27 serum-free supplement (Life Technologies, Paisley, UK). Half medium changes were performed every 2–3 days and cultures were differentiated for up to 61 days (days differentiated (DD) 61).

### Transfection

Differentiated CTX0E16 neurons were transfected at the desired age using Lipofectamine 2000 (Life Technologies, Paisley, UK) as previously described [22]. Briefly, 2 μg of peGFP-C1 was mixed with 2 μl of Lipofectamine 2000 and incubated for 20 minutes in a humidified atmosphere of 95 % air/5 % CO2 at 37 °C with open caps. The DNA:Lipofectaimine 2000 mixture was added dropwise to CTX0E16 neurons, and cells were incubated for 4 hours at 37 °C, before being transferred to new wells containing fresh media. Transfections were allowed to proceed for 2 days before either treatment or fixation and subsequent immunocytochemical analysis.

### Immunocytochemical analysis and microscopy

Prior to fixing, CTX0E16 cells were washed once in warm PBS. Cells were then fixed in either 4 % formaldehyde/4 % sucrose in PBS at room temperature for 20 minutes, or in 4 % formaldehyde/4 % sucrose in PBS at room temperature for 10 minutes followed by two washes with PBS and incubation with pre-chilled (−20 °C) methanol for 10 minutes at 4 °C. Methanol fixation was used to unmask protein in lipid rich structures, such as the post-synaptic density [22]. Cells were washed a further two times in PBS and then blocked in PBS containing 30 % normal goat serum with 0.1% Triton X-100 (Sigma, St. Louis, MO, USA) for 2 hours at room temperature. Primary antibodies were added in PBS containing 10 % normal goat serum overnight at 4 °C, followed by 3 × 15-minute washes in PBS. Cells were then incubated for a further hour in 10 % NGS with 0.1 % Trinton X-100, before incubation with Alexa, 488, 568 or 633 secondary antibody (Life Technologies, Paisley, UK) diluted in PBS with 10 % normal goat serum for one hour at room temperature. Two further washes (15 minutes each) were performed before coverslips were washed once with PBS containing 0.1 % 4′,6-diamidino-2-phenylindole (DAPI), and then mounted using ProLong antifade reagent (Life Technologies, Paisley, UK). A list of antibodies used in this study can be found in Additional file 1: Table S1.

For some conditions, representative images were taken on a Zeiss Axio Imager Z1, equipped with an ApoTome and an AxioCam MR3 camera running AxioVision 4.7.1 imaging software (Carl Zeiss, Jena, Germany). Images of double- and triple-labelled neurons were obtained using a Lecia SP5 confocal microscope and × 63 objective (N.A. 1.4). Images were taken as a z-series (z-step = 0.4 μm). All images were exported to ImageJ [23] where maximum intensity projections and background subtracted images were generated [22]. In the green/purple colour scheme, co-localization is indicated by white. Images were taken in the linear range, to allow an accurate representation. Each channel is shown individually in greyscale.

Cell counts were performed using images acquired with either a Zeiss Axioimager or Olympus IX70 with × 20 objective (N.A. 0.8), with at least four fields of view (FOV) imaged per biological replicate per condition. Three biological replicates were imaged for each staining condition at each of the time points assessed, with one to two technical replicates for each. Z-stacks of four slices at 1 μm between each slice were Z-projected and the resulting projections were background subtracted. In all conditions, the DAPI channel was thresholded and regions of interest (ROI) were created around each nucleus and used to assess expression of MAP2 along with the various markers of cell identity via the average intensity of each area in the respective channels. The percentage of cells expressing each marker in each biological replicate was calculated relative to the total number of cells (DAPI-positive cells) and the number of MAP2-positive cells. These percentages for each biological replicate were then averaged and the standard errors calculated. All image processing for the quantification of cell counts was performed using ImageJ, and all subsequent analysis of these data was performed using Microsoft Excel.

### RNA preparation, cDNA synthesis, reverse transcription PCR (RT-PCR) and quantitative PCR (q-PCR)

Proliferative and differentiated (28 days) CTX0E16 cells were pelleted and lysed using TRIzol reagent (Life Technologies, Paisley, UK) and total RNA extracted according to the manufacturer’s instructions. Residual genomic DNA was removed from each of four biological replicates using the TURBO DNA-*free*™ Kit (Life Technologies, Paisley, UK) according to published protocols. cDNA was synthesized from 1.5 μg of total RNA from each extraction using random decamers and SuperScript III (Life Technologies, Paisley, UK), according to the manufacturer’s instructions.

To determine the expression of specific genes, primers were designed to target all known RefSeq transcripts of genes of interest, sourced from the University of California Santa Cruz (UCSC) Genome Browser website [24] (Additional file 1: Table S2). Where possible, primers were designed to span intronic regions of the selected genes to ensure specific amplification of mRNA, even in the presence of DNA contamination. Reactions were carried out in a total volume of 20 μl containing diluted cDNA, 1 × HOT FIREPol Blend Master Mix (Solis Biodyne, Tartu, Estonia) and primers at 200 nM, using a GS4 thermal cycler. Samples were separated and visualised by polyacrylamide gel electrophoresis.

For quantitative expression analysis, 20 μl cDNA samples from SuperScript III reactions were diluted with a further 120 μl of nuclease-free H_2_O. Reactions were carried out in a total volume of 20 μl, containing diluted cDNA, 1 × HOT FIREPol® EvaGreen® q-PCR Mix (Solis Biodyne, Tartu, Estonia) and primers at 200 nM, using an MJ Research Chromo 4 (Bio-Rad, Hercules, CA, USA) and MJ Opticon Monitor analytic software (Bio-Rad, Hercules, CA, USA). Triplicate q-PCR reactions were performed to measure each gene in each cDNA sample. The level of each gene was measured against a standard curve constructed by serial dilution of pooled cDNA from all assayed samples. A relative value was thus obtained for each of the three triplicate reactions for each cDNA sample. Mean measures of target genes were then normalized against a geometric mean determined from three internal control genes (GAPDH, HPRT1 and RPL13A) for each cDNA sample to yield a relative target gene expression value for all samples. GAPDH, HPRT1 and RPL13A were identified as suitable internal controls on the basis of previous whole-genome microarray data of CTX0E16 cells, where it showed the least variability (in terms of standard deviation) across conditions. Normalized q-PCR target gene expression values were compared between proliferative (DD 0) or DD 28 CTX0E16 cells.

### Calcium imaging

For experiments to assess responsiveness of CTX0E16 cells to exogenous neurotransmitter ligands, cells were differentiated for 28 days (DD 28) on PDL (5 μg/ml) and laminin-coated (1 μg/cm^2^) glass coverslips, and then incubated with HEPES-buffered physiological saline solution (140 mM NaCl, 5 mM KCl, 2 mM CaCl_2_, 1 mM MgCl_2_, 10 mM HEPES and 10 mM glucose, buffered to pH 7.4 using 5 M NaOH) supplemented with 2.5 μM Fura-2 AM and 1 mM probenecid (loading buffer) at 37 °C for 60 minutes before being replaced with assay buffer (loading buffer minus Fura-2 AM and probenecid). Images of a group of cells were taken every 2 seconds with × 10 objective at the 520 nm emission wavelength following excitation at 340 and 380 nm using a microscope-based imaging system (Photon Technology International, Birmingham, NJ, USA). Each experiment began with 2 minutes of baseline readings to allow cells to settle before the addition of the neurotransmitter compound being tested (dopamine, glutamate or acetylcholine; Sigma, St. Louis, MO, USA) at 1 mM, diluted in HEPES-buffered physiological saline solution, to which cells were exposed for one minute. Image analysis of individual cells by emission intensity ratios from 340 nm/380 nm excitation were performed, using the ImageMaster software (Photon Technology International, Birmingham, NJ, USA).

To investigate spontaneous Ca^2+^ transients in CTX0E16 neurons, cells were differentiated for 42 days on coverslips, then loaded with 2 μM Fluo-4-AM dye (Life Technologies, Paisley, UK) in HEPES-buffered physiological saline solution (140 mM NaCl, 5 mM KCl, 2 mM CaCl_2_, 1 mM MgCl_2_, 10 mM HEPES, 10 mM glucose and buffered to pH 7.4 using 5 M NaOH) supplemented with 0.02 % Pluronic-F27 (Life Technologies, Paisley, UK) for 15 minutes at 37 °C, followed by washing in HEPES-buffered physiological saline solution for a further 15 minutes at 37 °C. Fluo-4 fluorescence was time-lapse-recorded (1 second intervals) with MetaMorph (Molecular Devices, Sunnyvale, CA, USA) on an Axiovert S100 microscope (Carl Zeiss, Jena, Germany) equipped with appropriate filter sets (Chroma Technology, Bellows Falls, VT, USA), a × 40 (N.A. 1.3) PlanNeofluar objective (Carl Zeiss, Jena, Germany) and a Photometrics Cascade-II 512B EMCCD. Indicated agonists, KCl (50 mM) and Tetrodotoxin (TTX; 1 μM), were diluted in HEPES-buffer and added to cells to give the final concentration. In all experiments, 10 μM ionomycin was added as a second stimulation for the dye loading control. Offline analysis of the intensity pattern of the Fluo-4 signal was performed in ImageJ. ROI were drawn around the soma of cells with clear dendritic processes. Spontaneous activity was categorized when at least one clear neuronal calcium event was detected on a soma. Neuronal calcium events were defined as a sharp transient increase in fluorescence intensity: Fluo-4, ΔF/F0 >5%, with a fast rise and slower decay.

### Electrophysiology

Electrophysiological recordings were obtained from CTXOE16 neurons differentiated for between 29 and 61 days. Patch pipettes (4.0–7.5 MΩ) were pulled from borosilicate glass capillary tubes using a P97 Flaming/Brown Micropipette Puller (Sutter Instruments). The internal patch solution contained (in mM) 135 KGluconate, 10 KCl, 1 MgCl_2_, 10 HEPES, 2 Na_2_-ATP and 0.4 Na_3_-GTP. All recordings were conducted at room temperature. The external recording solution contained (in mM) 139 NaCl, 2.5 KCl, 10 HEPES, 10 Glucose, 2 CaCl_2_ and 1.3 MgCl_2_. For recordings in older cells (DD 55−DD 61) electrophysiological recordings were conducted in NDM. Whole cell voltage clamp recordings were conducted at a holding potential of −70 mV to monitor predominantly α-amino-3-hydroxy-5-methyl-4-isoxazolepropionic acid (AMPA) receptor-mediated spontaneous excitatory post synaptic currents (EPSCs) and at +40 mV to monitor EPSCs mediated by N-methyl-D-aspartate (NMDA) receptors. A stable access resistance was maintained between 20–35 MΩ for the duration of these recordings with no series resistance compensation. In some cases, tight-seal cell-attached recordings were conducted in order to monitor spontaneous action potential firing. In all experiments, corrections were made for liquid junction potentials offline. Data were generated and acquired using an EPC10 amplifier (Heka Instruments, Bellmore, NY, USA) and the software PatchMaster. Data were sampled at 15 kHz and filtered at 3 kHz.

### ERK1/2 phosphorylayion assay

The ability of neurotransmitter compounds (dopamine, glutamate or acetylcholine; Sigma) to activate Extracellular signal-regulated kinases 1, 2 (ERK1/2) in differentiated (DD 28) CTX0E16 neurons, was assessed using the Cellul’erk Kit (Cisbio Bioassays, Codolet, France). Cells were plated onto laminin-coated (0.5 μg per well) clear 96-well plates at a density of 30,000 cells per well. Cells were incubated with the neurotransmitter compounds to be tested for 0–60 minutes. Neurons were then lysed in lysis buffer supplemented with blocking reagent and incubated for 5 minutes at room temperature with shaking. Cell lysates from each well were then transferred to wells of a 384-well, solid bottom, small volume, white plate (Greiner, Stonehouse, UK) and mixed with 2 μl of each of the two HTRF conjugates (anti-ERK1/2-Eu^3+^-cryptate (donor) and anti-phospho-ERK1/2-d2 (acceptor)) before being incubated for 2 hours at room temperature. Each 384-well plate additionally contained 6 wells dedicated to negative (16 μl of blocking reagent-supplemented lysis buffer with 2 μl of anti-ERK1/2-Eu^3+^-cryptate donor conjugate and 2 μl of detection buffer - donor antibody only) and blank (16 μl of blocking reagent-supplemented lysis buffer with 2 μl of each of the donor and acceptor conjugates) controls, respectively. Plates were then read using a FlexStation 3 or Artemis K-101 plate-reader. Readings were made at 620 nm for the donor (Eu^3+^-cryptate) and 665 nm for the acceptor (d2) following excitation at 314 nm. Readings were an average of 50 replicates with a delay and integration time of 100 μ seconds. A 665 nm/620 nm ratio was calculated for each well/assay and referred to as *R*. A mean *R* value was calculated for the 6 negative control wells on each 384-well plate and referred to as *R*_*Neg*_. Values were expressed as *ΔF*% according to the following equation as defined by Albizu and colleagues [25]:

$$ \varDelta F\%=\frac{R-{R}_{Neg}}{R_{Neg}}\times 100 $$

*ΔF*% values were analysed using GraphPad Prism.

### Neuronal treatment: chronic neuronal depolarisation

DD 13 CTX0E16 neurons were transfected with enhanced green fluorescent protein (eGFP) as described previously. At DD 15, media were replaced with warm experimental media for each of the three experimental conditions: NDM supplemented with pure water, NDM supplemented with 30 mM NaCl as an osmotic control or NDM supplemented with 30 mM KCl. Cultures were fixed and immunostained after 7 hours of exposure to the experimental media. Cells were imaged using a Zeiss fluorescence microscope with × 63 objective (N.A. 1.4). Only cells with a neuronal morphology and more than one neurite were imaged for the purpose of assessing neurite length. Images were processed using ImageJ and neurites were traced using the NeuronJ plugin. Imaging and neurite tracing was carried out with the experimenter blinded to experimental condition.

### Statistics

**Fig. 1:**
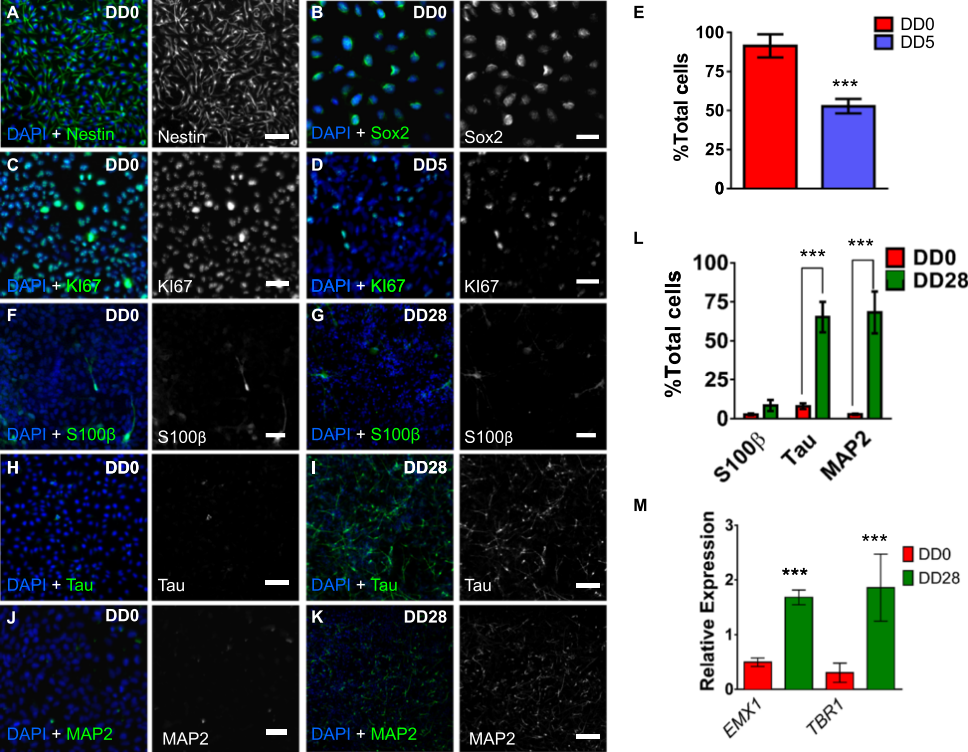
Characterisation of undifferentiated and differentiated CTX0E16 hNPCs. **a**, **b** Representative images of undifferentiated (days differentiated (*DD*) 0) CTX0E16 cells immunostained for the neural progenitor cell (NPC) markers nestin (**a**) and Sox2 (**b**). **c** At DD 0, the majority of CTX0E16 NPCs were positive for the cell proliferation marker, KI67. **d** By DD 5, the number of KI67-positive CTX0E16 cells had greatly reduced. **e** Quantification of the number of KI67 positive cells as a percentage of total cells; n = approximately 1,500 cells from three independent experiments carried out in triplicate; *error bars* represent SD; ***p <0.001 (Student’s unpaired *t* test). **f**, **g** Only a few cells were positive for the astrocyte marker S100β at DD 0 (**f**) or DD 28 (**g**). **h**, **i** At DD 0 very few cells expressed the neuronal marker Tau (**h**). However, after 28 days of differentiation, the majority of cells expressed Tau (**i**). **j**, **k** Similarly, very few cells were positive for the neuronal marker MAP2 at DD 0 (**j**), but by DD 28 the majority of cells were positive for MAP2, indicating that the vast majority of cells at this time point had differentiated into neurons. **l** Number of proliferative (DD 0) or differentiated (DD 28) cells positive for S100β, Tau or MAP2; N = approximately 1,500 cells from three independent experiments carried out in triplicate; *error bars* represent SD; ***p <0.001 (two way analysis of variance with Tukey post hoc analysis). **m** Quantitative PCR (q-PCR) analysis revealed an increase in the expression of dorsal forebrain marker, *EMX1* and cortical neuron marker *TBR1* in DD 28 CTX0E16 neurons compared to DD 0 cells; n = 4 independent experiments carried out in triplicate; *error bars* represent SD; ***p <0.001 (Student’s unpaired *t* test). Scale bar, 50 μm. *DAPI* 4′,6-diamidino-2-phenylindole

**Fig. 2:**
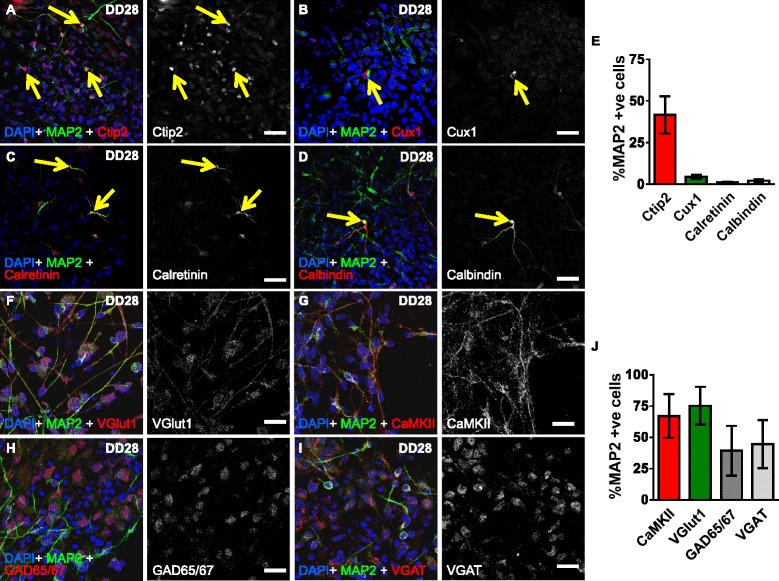
Differentiation of CTX0E16 cells generates glutamatergic cortical neurons. **a**-**d** Representative images of differentiated (DD 28) CTX0E16 neurons co-stained for 4′,6-diamidino-2-phenylindole (DAPI), MAP2 and either the deep cortical layer marker Ctip2 (**a**), the upper cortical layer marker Cux1 (**b**), GABAergic interneuron marker calretinin (**c**) or calbindin (**d**). *Yellow arrows* indicate neurons expressing these markers. **e** Quantification revealed that 41.6 % of MAP2-positive neurons expressed Ctip2, whereas less than 10 % of MAP2-labelled neurons were positive for Cux1, calretinin or calbindin; n = approximately 1,500 cells per condition from three independent experiments carried out in triplicate; *error bars* represent SD. (**f**, **g**) Images of DD28 CTX0E16 neurons co-stained for DAPI, MAP2 and the glutamatergic and excitatory markers VGlut1 (**f**) or CaMKIIα (**g**). **h**, **i** DD 28 CTX0E16 neurons were also co-stained for DAPI, MAP2 and the GABAergic markers GAD65/67 (**h**) or VGAT (**i**). **j** Quantification of F-I revealed that over 65 % of MAP2-psoitive neurons also expressed VGlut1 or CaMKIIα, whereas less than 45 % of MAP2-neurons were also positive for either GAD65/67 or VGAT; n = approximately 1,500 cells per condition from three independent experiments carried out in triplicate; *error bars* represent SD. Scale bar, 20 μm

For all experiments, two to four technical replicates from three to four independent cultures/passages were used. For all graphs, bars represent means and error bars as either SD (Figs. 1 and 2) or standard error of the mean (SEM). To identify differences among conditional means, statistical analyses (Student’s unpaired *t* test, analysis of variance (ANOVA)) were performed in either Excel or GraphPad Prism 5. Tukey post hoc analysis was used for multiple comparisons.

## Results and discussion

### CTX0E16 cells differentiate into neurons of distinct lineages

A number of immortalised hNPC lines generated from human fetal ventral telencephalon, spinal cord or cortex have previously been described. These lines were conditionally immortalised using either a c-myc or v-myc transgene, and have been shown to differentiate into specific neural cell types under specified neural growth mediums [6, 11–16]. Therefore, we initially sought to determine whether differentiated CTX0E16 NPCs were capable of adopting a neuronal fate. Proliferative (DD 0), CTX0E16 NPCs were routinely maintained as a monolayer in RMM for up to 30 passages. As expected these cells expressed the intermediate filament protein nestin and Sox2 neural precursor markers, indicative of an NPC phenotype (Fig. 1a, b). Proliferative CTX0E16 cells also highly expressed KI67, a marker of cell proliferation; however, the number of KI67-positive cells was significantly reduced 5 days after cells were cultured in NDM (percent total cells positive for KI67: DD0, 91.4 ± 3.69; DD5, 52.8 ± 2.31; *p* <0.001, Fig. 1c-e). This indicated that proliferation of CTX0E16 cells was rapidly halted following initiation of differentiation.

We next examined the neural cell type into which CTX0E16 cells differentiated. Under proliferative conditions less than 10 % of cells were positive for the astrocyte and ependymal cell marker, S100β, or the neuronal markers Tau or MAP2 (Fig. 1f-l). Conversely, after 28 days of differentiation (DD 28) there was a marked increase in the number of Tau and MAP2 positive neurons; no change in S100β-positive cells was observed (Fig. 1f-l). These data indicate that differentiation of CTX0E16 NPCs resulted in the generation of cells with a neuronal fate.

Previously characterised hNPCs have been shown to retain an identity associated with the region from which they were derived [5, 11, 12, 14–16]. In addition, studies using mouse or human embryonic stem cells, or human induced pluripotent stem cells (hiPSCs), have shown that withdrawal of mitogens and culturing in the presence of factors associated with generation of telencephalon/forebrain cells, results in the production of glutamatergic projection neurons of all cortical layers [17, 26, 27]. Thus, we reasoned that differentiated CTX0E16 neurons would express markers of cortical identity. Consistent with this, q-PCR analysis revealed a significant increase in the dorsal forebrain marker *EMX1*, and the cortical marker *TBR1* 28 days after differentiation, indicating that CTX0E16 NPCs were differentiating into neurons with a cortical identity (Fig. 1m). In vivo, a fundamental characteristic of cortical stem cells is the generation of neurons into layer-specific neuronal subtypes, as determined by the expression of a number of specific molecular markers [17, 26, 27]. Therefore, to examine whether differentiated CTX0E16 cells generated a diverse population of cortical neurons, we assessed whether they expressed specific cortical layer markers. Accordingly, we found that 41.6 % of MAP2 neurons were positive for Ctip2 (Fig. 2a, e), a marker of deep-layer cortical neurons, consistent with the observed increase in *TBR1* expression. Conversely, less than 10 % of MAP2 neurons were positive for the upper layer marker Cux1 (Fig. 2b, e). Next we examined whether any subtypes of GABAergic interneurons were present in our cultures. Interestingly, less than 10 % of MAP2 neurons were calretinin-positive or calbindin-positive cells (Fig. 2c-e); no parvalbumin-positive cells were detected. One possible explanation for the lack of parvalbumin interneurons in our cultures is that this subtype of GABAergic interneurons is only observed at a later point during the development of the neocortex [11, 28, 29]. Nevertheless, these data indicate that the majority of neurons generated by CTX0E16 NPCs are pyramidal neurons. Consistent with this observation, 67.1 ± 10.15 % of MAP2 neurons expressed the glutamatergic and pyramidal marker Vesicular glutamate transporter 1 (VGlut1, also known as SLC17A7) and 75.2 ± 8.74 % of MAP2 neurons were positive for excitatory neuronal marker CaMKIIα (Fig. 2f-g, j). Both VGlut1 and CaMKII were detected in the cell soma, and along processes in puncta structures, suggesting that at least some of these proteins may be present at synapses. Interestingly, VGlut2 has been found to be present in the cytoplasm of immature mouse neurons and in immature neurons derived from mouse ESCs [30, 31]. It has been suggested that during early development, and in particular during axogenesis or synaptogenesis, neurons produce high levels of synaptic proteins, such as VGlut2. Therefore, the presence of VGlut2, may result from an accumulation of protein in the cell body waiting to be transported out along axons [31]. Thus, it is also possible that the presence of VGlut1 in the cell soma of DD 28 CTX0E16 may be indicative of accumulated protein waiting to be transported out along axons to newly formed synapses. Remarkably, a significant number of CTX0E16 neurons also expressed the GABAergic marker GAD65/67 (39.3 ± 11.5 %) (Fig. 2h, j) suggesting that CTX0E16 may be forming inhibitory synapses. In agreement with this, a subpopulation of CTX0E16 neurons (44.6 ± 11.1 %) also displayed punctate staining for the vesicular GABA transporter (VGAT). Indeed, VGAT immunoreactivity was found mainly around the cell soma (Fig. 2h-j), indicating that these cells were potentially receiving GABAergic inputs. The relatively high expression of inhibitory markers in contrast to the low number of interneurons could be explained by the fact that GABAergic synapses are functionally excitatory during development [32]. Critically, these data suggest that, of the neurons generated, the majority of cells display characteristics of glutamatergic projection (cortical) neurons, with only a few GABAergic interneurons being generated.

Collectively, these data indicate that CTX0E16 cells are multipotent and can differentiate into distinct neural sub-types. While this includes a small number of astrocytes and GABAergic interneurons, the majority of neurons generated express a subset of molecular makers consistent with the formation of glutamatergic cortical neurons of distinct cortical layers. Thus, our data suggest that as NPCs generate neurons of a fate synonymous with their region of origin.

**Fig. 3:**
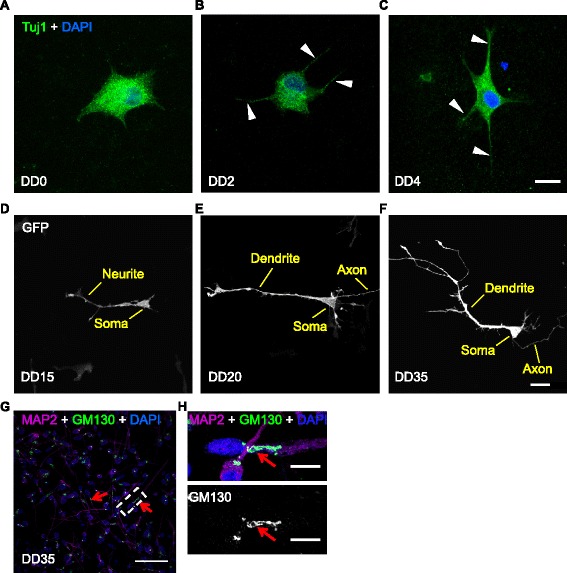
Differentiated CTX0E16 neurons display morphological features of pyramidal neurons. **a**-**c** Generation of small neurites in βIII Tubulin (*Tuj1*)-positive CTX0E16 cells after 2 or 4 days of differentiation. **d**-**f** Expression of green fluorescent protein (*GFP*) in young CTX0E16 neurons, reveals the development of neuronal morphology; at differentiation day (*DD*) 15, neurite processes extend from the cell’s soma (**d**). By DD 20, a single long and thin process can be seen emerging from the cell’s soma with a thicker single process also emerging from the opposite side. Note the pyramidal shape of the cell soma. **f** By DD 35, the dendritic process displays some level of arborisation; additional smaller processes protruding from the cell soma are also evident. **g**, **h** Double immunostaining of DD 35 CTX0E16 neurons for the trans-Golgi marker, GM130 and MAP2. In MAP2-positive neurons, GM130 is clearly seen orientated towards a single, typically the longest, dendrite (*red arrows*). This indicates the primary dendrite, and the formation of a polarized morphology (**g**). **h** High-magnification images reveal that the Golgi-network is present along the primary dendrites (*red arrow*). Scale bars, 20 μm (**a**-**g**) and 5 μm (**h**). *DAPI* 4′,6-diamidino-2-phenylindole

Differentiated CTX0E16 neurons exhibit typical pyramidal neuronal morphology. An important characteristic of cortical glutamatergic neurons is their ability to generate unipolar pyramidal neuronal morphology: interneurons display multipolar morphologies [17, 33, 34]. In vivo this is typified by the generation of unipolar neurons with a single apical dendrite that projects to the pial surface [35–37]. In vitro, primary rodent neuronal cultures have also been shown to generate unipolar neurons, with a distinct primary dendrite (akin to apical dendrites in vivo) [33, 36–38]. Moreover, the generation of this polarized morphology in vitro can be divided into several distinct stages: 1) generation of small neurite processes; 2) specification of a single axonal process; 3) formation of dendrites from remaining neurites; 4) maturation of axonal and dendritic processes and formation of synaptic connections [33]. To investigate the development of neuronal morphologies in differentiated CTX0E16 neurons, cells were fixed at early stages of differentiation (DD 0, DD 2 and DD 4) and immunostained for βIII Tubulin (Tuj1), a marker of young neurons (Fig. 3a-c). After two days of differentiation, CTX0E16 cells had begun to extend neurite-like processes (Fig. 3b, arrow heads), while by day 4 of differentiation the amoeboid morphology of the NPCs was found to have developed into a more neuronal-like appearance, with cells exhibiting multiple neurites (Fig. 3c, arrow heads). In order to examine the morphology of neurons differentiated for longer periods of time, we transfected cells with eGFP to outline cell morphology. By DD 15, CTX0E16 neurons had adopted a polarised neuronal morphology; the extension and arborisation of a single process, characteristic of the specification of an axonal process, could be clearly identified (Fig. 3d). After 20 days of differentiation, maturation of the axon had occurred, and the formation of a primary dendrite was evident (Fig. 3e). By DD 35, CTX0E16 neurons had formed dendrites with secondary and tertiary dendritic arborisations, indicative of a young, yet maturing pyramidal neuron (Fig. 3f). Numerous studies have demonstrated that cultured rodent cortical neurons develop a typical polarised morphology with primary and non-primary dendrites: primary dendrites resemble apical dendrites in vivo, while non-primary dendrites resemble basal dendrites, as seen in vivo [36, 37, 39]. We find that after 35 days of differentiation, a single long dendrite, with several branches, can be observed. This is likely to be the primary dendrite. Very few non-primary dendrites were seen at DD 35, however, this is likely due to the fact that *in vivo*, basal dendrites are formed after the initial establishment of the apical dendrites [35], a phenomenon that is recapitulated in vitro [36].

Another hallmark of the generation of morphologically accurate cortical neurons in vivo is that the trans-Golgi network can also be seen in the apical dendrite [36, 38–40]. Interestingly, in primary neuronal cultures, the trans-Golgi network has been shown to extend into the primary dendrite, consistent with the formation of a polarised morphology [36, 38–40]. Thus, to further confirm whether CTX0E16 neurons generated polarised neuronal morphologies, we stained DD35 cultures for the dendritic marker MAP2 and the *cis*-Golgi network protein, GM130. The majority of MAP2-positive neurons had discernible GM130 staining within the soma (Fig. 3g). Interestingly, high magnification close-ups demonstrated that in the majority of MAP2-positive CTX0E16 neurons, GM130 was orientated towards a single MAP2-process, typically the longest process, indicative of the formation of a polarised neuronal morphology (Fig. 3h). Moreover, GM130 was occasionally seen to extend into a single dendrite, consistent with the formation of a primary dendrite, but also of a secretory network indicative of a functional neuron (Fig. 3h). Critically, these findings further indicate that differentiated CTX0E16 cells develop neurons with a morphology and polarisation consistent with that associated with glutamatergic pyramidal neurons.

### Expression of neurotransmitter receptor, signalling proteins and disease-related proteins

**Fig. 4:**
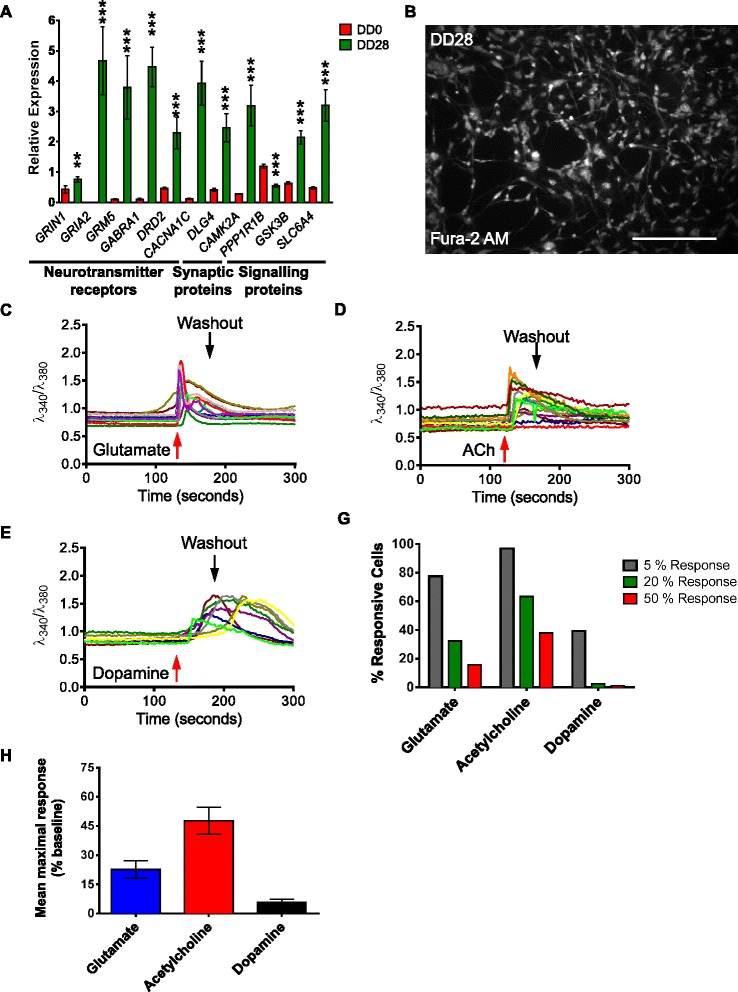
CTX0E16 cells generate neurons that respond to neurotransmitter stimulation. **a** Expression of a subset of neurotransmitter receptors, synaptic and signalling proteins is increased in differentiation day (*DD*) 28 CTX0E16 neurons as compared to undifferentiated (DD 0) CTX0E16 neural stem cells (NPCs) indexed by quantitative PCR (q-PCR); n = 4 independent experiments carried out in triplicate; *error bars* represent SD; **p <0.01; ***p <0.001 (Student’s unpaired *t* test). **b** Representative image of neurons loaded with Fura-2 AM used for single cell Ca^2+^ imaging. **c**-**e** Representative traces of intracellular Ca^2+^ in response to various neurotransmitter receptor ligands. **g** Number (%) of total cells generating 5 %, 20 % or 50 % responses following treatment with neurotransmitter ligands; n = 3 independent experiments carried out in triplicate (600 cells in total). **h** Mean maximal response produced by DD 28 CTX0E16 neurons following application of neurotransmitter ligands; n = 3 independent experiments carried out in triplicate (600 cells in total); *error bars* represent SD

The ability of CTX0E16 NPCs to differentiate into neurons with a mainly glutamatergic phenotype led us to question whether these cells also express a range of neuronal genes (Additional file 1: Table S2). Using RT-PCR, proliferative CTX0E16 NPCs were found to express a select number of neurotransmitter-targeted G-protein-coupled receptors (GPCRs) and ionotropic receptor subunits, including those belonging to glutamatergic, dopaminergic, serotonergic and cholinergic receptor families, and calcium channels (Additional file 1: Figure S1). Expression of signalling hub molecules GSK-3β (*GSK3B*), DARPP-32 (*PPP1R1B*) and *SLC6A4*, and the adaptor proteins, *ARRB2*, *CAV1*, *DLG4* and *MPDZ*, were also detected in CTX0E16 NPCs. Following 28 days of differentiation, expression at the mRNA level was observed for all genes evaluated (Additional file 1: Figure S1). Robust expression was observed for AMPA glutamate receptor subunit *GRIA1* and the NMDA glutamate receptor subunit *GRIN1*, and for the metabotropic glutamate receptors, *GRM1*–*GRM8* (Fig. 4a). In addition, DD 28 CTX0E16 neurons also expressed the GPCRs, *ADRA1A*, *DRD2*, *DRD3* and *HTR2A*. Lower expression levels were seen for *CHRM1*, *HRH1*, *TACR3* and *AVPR1A*. The accessory proteins *ARRB2*, *CAV1*, *DLG4* and *MPDZ* and the 5-HT transporter, *SLC6A4*, were also found to be expressed (Additional file 1: Figure S1). We complemented these studies by analysing the change in expression level of a subset of key neurotransmitter receptor, synaptic and signalling protein genes, after 28 days of differentiation. Consistent with our characterisation of neuronal lineage, DD 28 neurons had significantly higher levels of the NMDA receptor subunit *GRIN1*, AMPA receptor subunit, *GRIA1*, in addition to elevated levels of *GABRA1* and DRD2. The synaptic proteins *DLG4* and *CAMK2A* and the voltage gated Ca^2+^ channel *CACNA1C* were also significantly elevated compared to DD 0 CTX0E16 NPCs (Fig. 4). Collectively, these data suggest that undifferentiated CTX0E16 cells express a range of neurotransmitter receptors and signalling proteins, which are upregulated during differentiation. Notably, this expression profile is consistent with differentiation along a glutamatergic cell fate.

### CTX0E16 neurons develop physiological characteristics

Based on our gene profiling and immunostaining data, CTX0E16 neurons would be expected to express a range of neurotransmitter receptors, adaptor, scaffold and key signalling proteins and a number of disease-relevant proteins (Figs. 2 and 4a and Additional file 1: Figure S1). Thus, in order to determine whether expression of a range of neurotransmitter receptor genes resulted in functional integration, we investigated the functional characteristics of CTX0E16 neurons using calcium imaging. At DD 28 CTX0E16 neurons were loaded with the Fura-2 AM ratiometric dye. Good cell loading of Fura-2 was demonstrated by CTX0E16 emissions at 520 nm following excitation at 380 nm (Fig. 4b). DD 28 CTX0E16 neurons exposed to 1 mM glutamate (Fig. 4c) elicited both fast, pronounced responses and slower responses of a lower magnitude. The fast, pronounced responses are likely mediated through fast-activated NMDA or AMPA receptors, while the slower responses of lower magnitude are attributable to the activation of metabotropic glutamate receptors [41]. Nearly 80 % of cells in differentiated CTX0E16 cultures responded to glutamate, though the responses from these cells were often modest, with just over 30 % of cells producing a response of 20 % above baseline and under 20 % producing responses in excess of 50 % above baseline (Fig. 4g). The average response from all measured cells was approximately 20 % above baseline level (Fig. 4h).

Acetylcholine potently and almost ubiquitously caused increased intracellular Ca^2+^ concentrations (Fig. 4d), with more than 60 % of cells producing responses of 20 % above baseline and 40 % producing responses of 50 % above baseline (Fig. 4g). The average increase in intracellular Ca^2+^ concentration in response to acetylcholine, almost 50 % above baseline, was more than double that seen in response to glutamate and an order of magnitude greater than responses provoked by dopamine (Fig. 4h). Traces from individual cells exposed to acetylcholine showed predominantly rapid and sustained Ca^2+^ accumulation, though some cells did demonstrate more gradual increases in Ca^2+^ levels. In contrast to the rapid Ca^2+^ accumulation observed in response to glutamate and acetylcholine, dopamine provoked much more gradual, and at times delayed, increases (Fig. 4e). This was expected due to dopamine exerting its actions through metabotropic receptors alone [42]. In addition to this, far fewer cells responded to dopamine with increases in intracellular Ca^2+^ concentrations. Less than 40 % of cells produced responses that were more than 5 % above baseline and less than 3 % produced responses that achieved levels of 25 % above baseline (Fig. 4g, h).

**Fig. 5:**
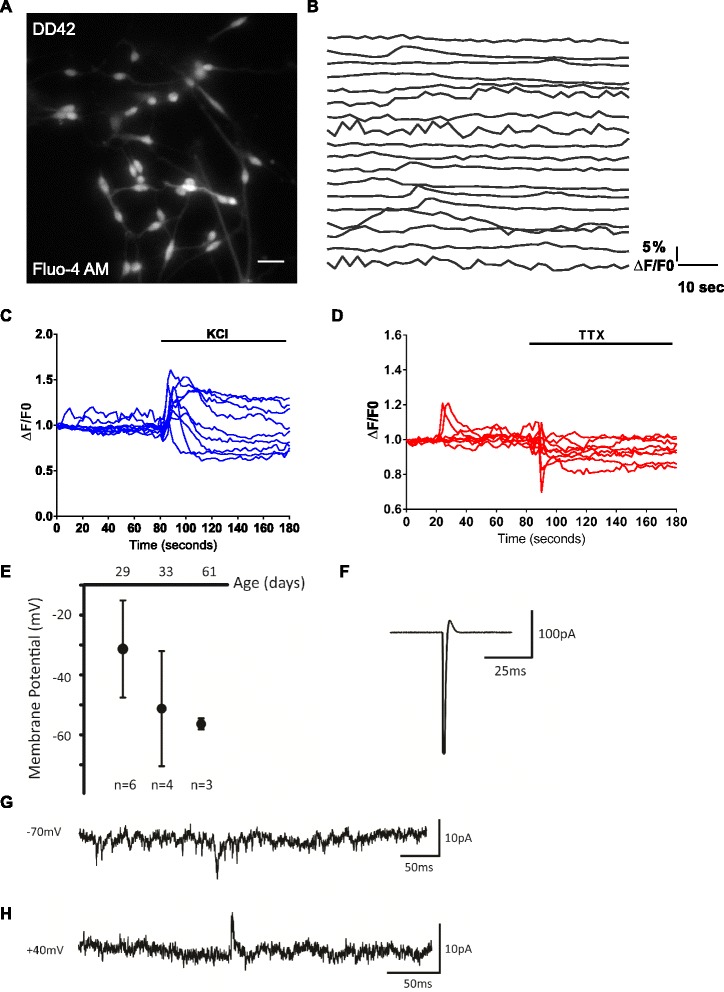
Development of functional properties in CTX0E16 neurons. **a** Representative image of Fluo-4 AM-loaded CTX0E16 neurons used for single cell Ca^2+^ imaging. **b** Representative time series of 18 neurons displaying spontaneous Ca^2+^ transients. Spontaneous activity was classified as a somatic calcium event greater than 5 % ΔF/F0: 38.0 ± 6.89 % cells displayed spontaneous activity over an 80-second period of imaging (n = 155 cells from 14 coverslips). **c**, **d** Representative traces of intracellular Ca^2+^ in responses to 50 mM KCl (**c**) or 1 μM tetrodotoxin (TTX) (**d**). **e** Resting membrane potential (Vm) recorded in current clamp progressively becomes more negative as CTX0E16 neurons become more mature (day of differentiation (*DD*) 29−DD 61); *error bars* represent SD. **f** Representative action potential recorded in voltage clamp in the cell attached configuration, recorded from DD 50 CTX0E16 neuron. **g** Representative voltage clamp recording at a holding potential of −70 mV in DD 36 CTX0E16 neurons. The downward deflections indicate the presence of AMPA receptor-mediated spontaneous excitatory postsynaptic currents (EPSCs). **h** Example of a spontaneous N-methyl-D-aspartate (NMDA) receptor-mediated EPSC recorded in voltage clamp at +40 mV from a DD 33 CTX0E16 neuron; n = 3–6 cells from at least three independent coverslips

A hallmark of the generation of neuronal cell types is the emergence of spontaneous Ca^2+^ oscillations due to neuronal activity [43, 44]. No spontaneous Ca^2+^ oscillations were observed in DD 28 CTX0E16 cultures. We therefore differentiated CTX0E16 cells for 6 weeks (DD 42) and performed single cell Ca^2+^ imaging using Fluo-4 (Fig. 5a). This revealed that 38.0 ± 6.89 % of CTX0E16 neurons displayed spontaneous activity, defined as a sharp transient Ca^2+^ flux in a neuronal cell soma (Fig. 5b). In addition, application of KCl, a depolarising agent, resulted in a large Ca^2+^ flux in 79.1 ± 4.2 % of DD 42 CTX0E16 neurons (Fig. 5c), indicating the presence of voltage-gated Ca^2+^ channels, consistent with the observed increase in *CACNA1C* expression levels upon differentiation (Fig. 4a). Furthermore, addition of 1 μm tetrodotoxin (TTX) blocked spontaneous Ca^2+^ transients (Fig. 5d).

As these data indicated that CTX0E16 neurons were developing physiological neuronal characteristics, we next performed whole cell patch clamp recordings on CTX0E16 neurons at different stages during development. This revealed an initially depolarised resting membrane potential that became progressively more negative with age (DD 29: −31.3 mV, n = 6; DD 33: −51.25 mV, n = 4; DD 61: −56.4 mV, n = 3), consistent with increasing neuronal maturation occurring over this period (Fig. 5e). Next we investigated the ability of CTX0E16 neurons to fire action potential discharges. Cell-attached voltage clamp recordings were conducted in order to record network activity without disturbing the intracellular milieu. The capacity of CTX0E16 neurons to fire single action potentials was only apparent from DD 50 (Fig. 5f). The ability of these cells to fire repetitively in bursts did not develop over the time-course of this study. However, it should be noted that recent studies examining the functional properties of human induced pluripotent stem cell (iPSC)-derived neurons have demonstrated that co-culturing with rodent astrocytes or primary neurons induces a faster maturation of physiological neuronal characteristics [45]. In the current study CTX0E16 were not co-cultured, and as such, co-culturing these cells with rodent astrocytes or primary neurons may accelerate the maturation of functional properties in these cells. In addition to investigating the ability of CTX0E16 neurons to fire action potentials, whole-cell voltage clamp recordings were conducted to record spontaneous EPSCs. By DD 33−36 a subset of CTX0E16 neurons displayed spontaneous EPSCs: predominantly AMPA receptor-mediated (holding potential −70 mV) (Fig. 5g) and NMDA receptor-mediated (holding potential +40 mV) (Fig. 5h) were observed, demonstrating the presence of functional excitatory synaptic connections. These findings are consistent with the generation of predominately excitatory neurons and an increase in the expression levels of AMPA and NMDA receptor subunits (Fig. 4a). Recordings were also conducted at 0 mV in order to isolate GABA-mediated inhibitory postsynaptic currents. However, no spontaneous postsynaptic currents were recorded at a holding potential of 0 mV, indicating a lack of functional inhibitory synapses in these cells. Taken together, these results indicate that CTX0E16 neurons develop physiological neuronal characteristics that become progressively more mature with time in culture.

### CTX0E16 neurons show increases in ERK1/2 phosphorylation in response to neurotransmitter receptor activation

**Fig. 6:**
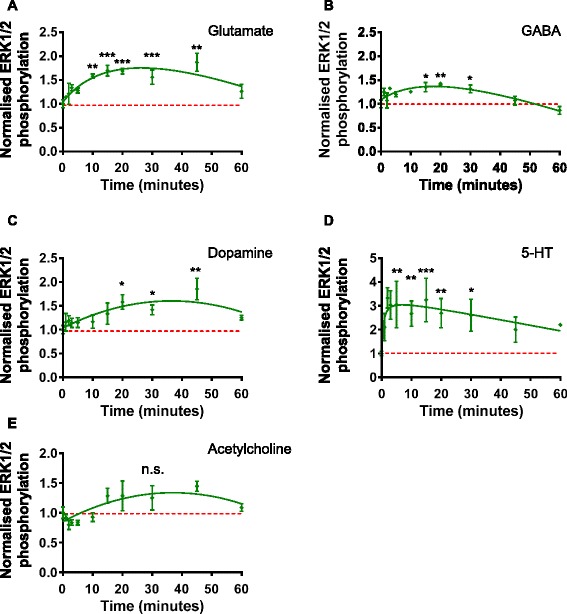
Phosphorylation of ERK1/2 kinase in differentiated CTX0E16 neurons following activation of neurotransmitter receptors. **a**, **b** Treatment with glutamate results in a time-dependent increase in phospho-ERK1/2 levels in differentiation day (*DD*) 28 CTX0E16 neurons. By 60 minutes, phospho-ERK1/2 levels are still raised as compared to control levels (**a**). Conversely, GABA application results in a transient increase in phospho-ERK1/2 levels; after 50 minutes, p-ERK1/2 levels have returned to baseline (**b**). **c**-**e** Treatment with dopamine (**c**) or 5-HT (**d**) results in activation of ERK1/2; CTX0E16 neurons respond to dopamine with a slower temporal profile as compared to 5-HT. Phospho-ERK1/2 levels are elevated following application of acetylcholine, but not significantly compared to baseline; n = 3 independent experiments carried out in triplicate; *error bars* represent SEM; *p <0.05; **p <0.01; ***p <0.001 (one way analysis of variance with Tukey post hoc analysis)

In addition to examining changes in Ca^2+^ levels in response to neurotransmitter activation, we further examined whether activation of these receptors led to the activation of intracellular signalling cascades. Owing to the ubiquity of ERK1/2 phosphorylation in response to neurotransmitter receptor activation and its many roles in neuronal function [46], this was used as a means to determine the presence of functional receptors in differentiated CTX0E16 cells. Glutamate, GABA, dopamine, 5-HT and acetylcholine were shown to induce ERK1/2 phosphorylation with different temporal profiles (Fig. 6). Glutamate induced a significant increase in ERK1/2 phosphorylation, peaking after 20 minutes, approximately 85 % above baseline, and gradually returning towards baseline within 60 minutes (Fig. 6a; *p* <0.01, ANOVA). GABA induced a more conservative, yet significant, phosphorylation of ERK1/2, again peaking at 20 minutes (approximately 40 % above baseline) before returning to baseline levels after 50 minutes (Fig. 6b; *p* <0.05, ANOVA). Application of dopamine resulted in a delayed increase in ERK1/2 phosphorylation, approximately 80 % after 40 minutes of exposure, and remaining elevated at 60 minutes (Fig. 6c, *p* <0.01, ANOVA). In contrast, a rapid increase in phosphorylated ERK1/2 levels were detected in response to 5-HT within 5 minutes, which reached approximately 3-fold above baseline levels (Fig. 6d, *p* <0.001, ANOVA). This was followed by a very gradual and linear decline that remained nearly 2-fold above baseline after 60 minutes. Acetylcholine evoked a modest, but insignificant increase in ERK1/2 phosphorylation levels (Fig. 6e). A very gradual increase in ERK1/2 phosphorylation was seen, resulting in an increase of approximately 30 % in ERK1/2 phosphorylation 20 minutes after stimulation (Fig. 6e). Taken together, these data demonstrate that CTX0E16 neurons express a range of neurotransmitter receptors that are capable of coupling to intracellular signalling cascades, further confirming the functional expression of various receptor systems.

### Expression of synaptic proteins in differentiated CTX0E16 cells

**Fig. 7:**
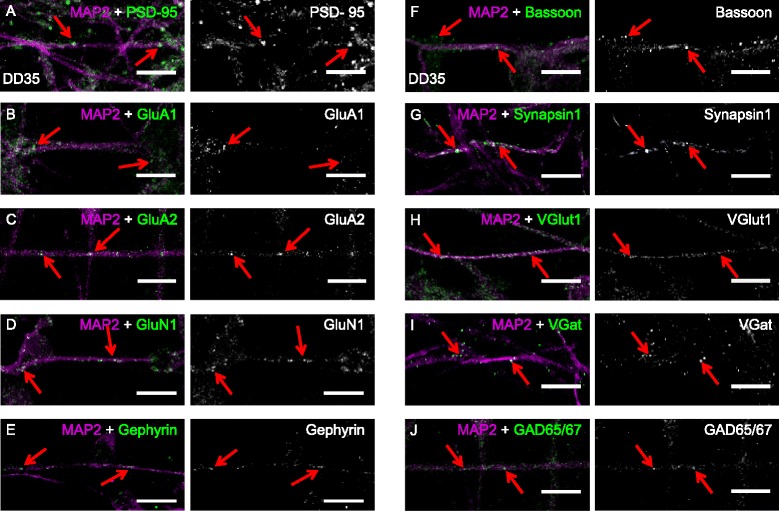
CTX0E16 neurons express pre- and post-synaptic proteins. **a**-**e** In differentiation day (DD) 35 CTX0E16 neurons, puncta for the post-synaptic proteins PSD-95 (**a**), GluA1 (**b**), GluA2 (**c**), GluN1 (**d**) and gephyrin (**e)** were observed along MAP2-positive dendrites (*red arrows*). **f**-**j** The excitatory pre-synaptic proteins bassoon (**f**), synapsin1 (**g**), VGlut1 (**h**), and the inhibitory pre-synaptic proteins VGAT (**i**) and GAD65/67 (**j**) were observed in punctate structures, either along or juxtaposed along MAP2-positive dendrites. Scale bars for **a**-**j** are 5 μm

Glutamatergic (excitatory) synapses comprise the majority of connections between pyramidal neurons in the mammalian forebrain. A defining characteristic of these synapses is the formation of specialised pre- and post-synaptic structures which contain a wide range of proteins [47]. This includes the presence of scaffold and glutamate receptor proteins on the post-synaptic side, as well as proteins associated with synaptic vesicle release or the uptake/metabolism of neurotransmitters on the pre-synaptic side. Previous investigations using cortically derived hNPC lines have only superficially demonstrated the presence of synaptic proteins or not at all [11, 14, 16]. As differentiation of CTX0E16 NPCs resulted in the generation of cells with a morphology associated with excitatory cortical neurons (Figs. 2 and 3), and that display electrophysiological properties characteristic of synaptic connections (Fig. 5), we investigated whether these cells also expressed pre- and post-synaptic proteins. By DD 35, immunoreactive puncta for the post-synaptic scaffold protein, postsynaptic density protein 95 (PSD-95, also known as SAP-90), could be seen along distal MAP2-postive dendrites in the majority of neurons (Fig. 7a). Consistent with being glutamatergic neurons, these cells exhibited punctate staining for the AMPA glutamate receptor subunits, GluA1 and GluA2 (Fig. 7b, c), as well as for the NMDA glutamate receptor subunit GluN1, along dendrites (Fig. 7d). Interestingly, gephyrin, a scaffold protein typically located at inhibitory synapses, was also observed in puncta structures along MAP2-positive dendrites, indicating the potential for the presence of inhibitory synapses (Fig. 7e).

We next investigated the presence of a range of pre-synaptic proteins. Punctate staining for the active zone marker bassoon and the synaptic vesicle protein synapsin 1 could be seen juxtaposed to MAP2-positive dendrites in DD 35 CTX0E16 neurons (Fig. 7f, g). The presence of these proteins indicates that the pre-synaptic machinery required for neurotransmitter release is present in differentiated CTX0E16 neurons. Furthermore, the glutamate transporter and excitatory synapse marker VGlut1 was also abundantly present along and next to MAP2-positive dendrites (Fig. 7h), consistent with the presence of excitatory synapses and post-synaptic glutamate receptors. Interestingly, the inhibitory pre-synaptic proteins VGAT (vesicular GABA transporter) and GAD65/67 were also present as punctate structures adjacent to dendrites (Fig. 7l, j) albeit at a low level. Taken together, these data demonstrate that differentiated CTX0E16 neurons express pre- and post-synaptic proteins indicative of the presence of both excitatory and inhibitory synapses.

### Pre- and post-synaptic proteins co-localise along dendrites in CTX0E16 neurons

**Fig. 8:**
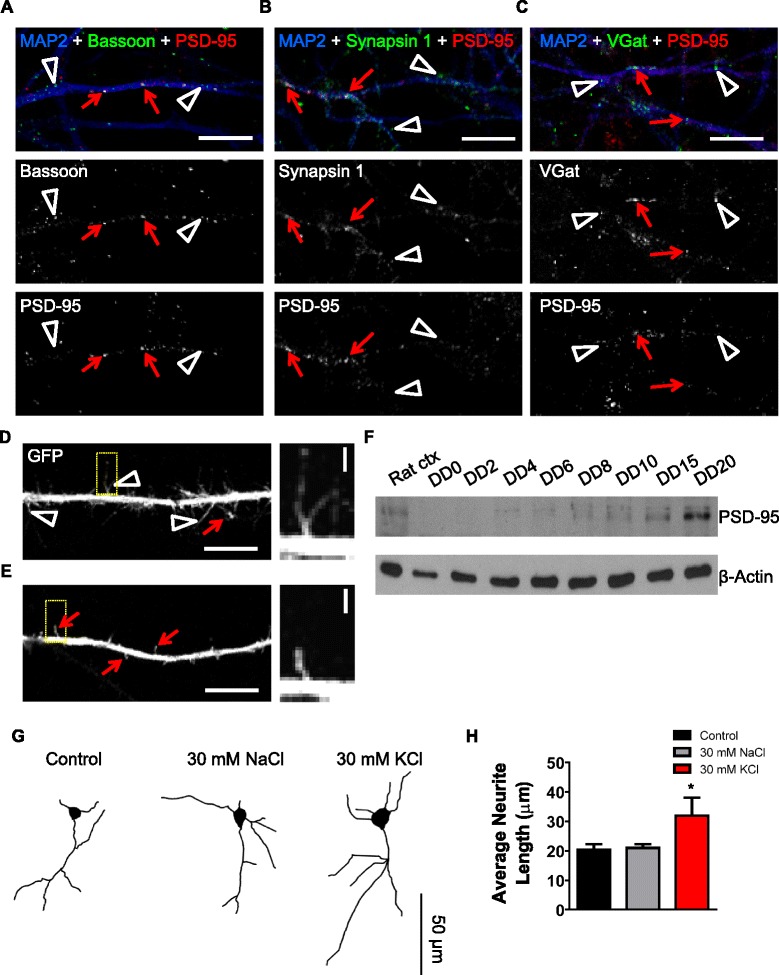
Differentiated CTX0E16 neurons display hallmarks of putative synapses and respond to activity-dependent stimulation. **a**-**c** Representative confocal images of differentiation day (DD) 35 CTX0E16 neurons immunostained for MAP2, PSD-95 and either bassoon (**a**), synapsin 1 (**b**) or VGAT (**c**). As previously observed, all synaptic proteins display punctate distribution along dendrites. In addition, a subset of PSD-95 puncta co-localised with all three pre-synaptic proteins (*red arrow*), indicating the presence of putative synapses; co-localisation is seen as white puncta. Not all pre-synaptic puncta co-localised with PSD-95 (*white open arrowheads*), suggesting that synaptogenesis was ongoing. **d**, **e** Representative confocal images of a distal dendrite of green fluorescent protein (GFP)-expressing ) CTX0E16 neurons (DD 35). Examination of dendrites revealed the presence of filopodia and dendritic spine-like structures, indicative of ongoing synaptogenesis. *Yellow boxes* indicate region used in high magnification in inset. **f** Western blotting of CTX0E16 neurons at different stages of differentiation demonstrate an increase in PSD-95 with maturation, consistent with an increase in synaptogenesis. Rat ctx, whole-cell lysate taken from rat cortex. **g** Representative binary images of GFP-expressing CTX0E16 neurons (DD 15) following treatment with control conditions (vehicle or osmotic control (30 nM NaCl)) or activity-dependent stimulation with 30 nM KCl for 7 hours. **h** Measurement of average neurite length in response to stimulation demonstrates that activity-dependent stimulation results in an increase in average neurite length; n = 14–22 neurons from 3–5 independent experiments carried out in triplicate; *error bars* represent SEM; *p <0.05 (one way analysis of variance with Tukey post hoc analysis). Scale bars for **a**-**e** are 5 μm; **d** and **e** inset, 1 μm; **g**, 50 μm

Synaptogenesis requires the coordination of a number of events, including the trafficking of pre- and post-synaptic proteins to specific locations where synapse formation can occur [48, 49]. Therefore, we sought to determine whether these puncta co-localised, thus indicating that CTX0E16 neurons were forming synaptic connections. In DD 35 CTX0E16 neurons a subset of PSD-95 puncta co-localised with the pre-synaptic and active zone marker, bassoon, along distal MAP2-positive dendrites (red arrows; Fig. 8a). However, not all bassoon and PSD-95 puncta co-localised with each other (white open arrowheads; Fig. 8a). This is likely due to the fact that synaptogenesis is an ongoing event during early stages of development, and the recruitment of both pre- and post-synaptic proteins to specific sites is required [48]. In a similar manner, PSD-95 and synapsin 1 also displayed partial co-localisation, with several puncta for each protein unopposed (Fig. 8b). While PSD-95 is predominately localised to excitatory synapses, it is also present at inhibitory synapses [47, 50]. Furthermore, recent evidence suggests that VGlut1 and VGAT may coexist at synapses, thus allowing for the release of both glutamate and GABA from select terminals [51]. Interestingly, we detected a small number of PSD-95 puncta that co-localised with VGAT in DD35 CTX0E16 neurons (Fig. 8c). This may suggest the presence of synaptic connections, although whether these are excitatory or inhibitory connections remains unclear as GABAergic synapses are thought to be functionally excitatory during development [32]. Moreover, the detection of overlapping synaptic proteins does not always indicate synaptic functionality.

During early postnatal development, synaptic dendritic protrusions first appear as long, thin, highly motile structures known as filopodia, which can initiate synaptic contacts with nearby axons [49, 52]. This initial contact between pre- and post-synaptic sides is a key step in synaptogenesis. The subsequent maturation and stabilisation of synapses is thought to involve filopodia transforming into highly specialised dendritic protrusions known as dendritic spines [49, 52]. In the mammalian forebrain, dendritic spines are the site where the majority of excitatory synaptic connections are located, and house the post-synaptic component of synapses [22, 47, 49, 52]. Based on our immunostaining data, we speculated that differentiated CTX0E16 neurons might display these dendritic protrusions. Detailed examination of distal dendrites of DD 35 CTX0E16 neurons expressing eGFP, as a morphological marker, revealed the presence of long, thin dendritic protrusions, with morphology consistent with that of filopodia (Fig. 8d). In a subset of neurons, smaller dendritic protrusions, occasionally defined by the presence of neck and head structures, were observed (Fig. 8e); such morphology is consistent with dendritic spines and maturation of synaptic connections. In support of this, western blotting of differentiated CTX0E16 neurons demonstrated an increase in expression of PSD-95 over time (Fig. 8f). Collectively, the pattern of juxtaposed pre- and post-synaptic proteins observed in differentiated CTX0E16 neurons and the presence of both filopodia and dendritic spines, strongly indicate that differentiated CTX0E16 neurons are capable of forming putative synapses.

### CTX0E16 neurons exhibit morphological plasticity in response to depolarisation

Early in development, the primary shape of a neuron and the identity of the emerging neurites as an axon or dendrites are progressively established. The regulated growth and arborisation of dendritic and axonal processes are critical to neural circuit formation, synapse formation and synaptic input processing [43, 53, 54]. Developing neurons are highly plastic and undergo modifications altering the branching pattern of neurites in response to physiological and pathological changes [43, 44, 53, 54]. It is now well-established that neuronal activity is a crucial modulator of neurodevelopment [43, 44, 53], and has a profound effect on neurite outgrowth as well as on dendritic and axonal formation and branching [44, 53, 54]. Interestingly, previous research has shown that prolonged depolarisation of neurons differentiated from human iPSCs results in an increase in the length of neurites via a Ca^2+^ -dependent mechanism [55]. Although our data indicated that differentiated CTX0E16 neurons expressed putative synapses, and exhibited functional responses to stimulation with a range of neurotransmitters, we questioned whether manipulating neuronal activity could regulate neurite outgrowth in these cells. This was achieved using a well-established protocol to induce neuronal depolarisation [55]: DD 15 GFP-expressing CTX0E16 neurons were exposed to control media, 30 nM NaCl (osmotic control) or 30 mM KCl for 7 hours, and the average length of neurites measured. This revealed that exposure to 30 mM KCl induced an increase of approximately 35 % in the average length of neurites compared to control or 30 mM NaCl conditions (average neurite length (μm): control, 20.46 μm ± 1.76 μm; 30 mM NaCl, 21.03μm ± 1.20 μm; 30 mM KCl, 31.95μm ± 6.02 μm; n = 12−22 cells, ANOVA, *p* <0.05; Fig. 8g, h). These data, taken together with the functional assays, strongly suggest that CTX0E16 neurons display the characteristics of young glutamatergic neurons that exhibit the functional characteristics of a developing synaptic network in vitro.

## Conclusion

The ability to model both normal and aberrant neurodevelopment in human neurons holds great potential for dissecting the underlying basic and disease-relevant mechanisms. Moreover, it provides another avenue for the discovery and screening of novel therapeutic molecules. Until recently a major challenge has been to efficiently produce a cost-effective and renewable source of cells that can reliably generate a defined set of neuronal cell types. Here we report that differentiation of the conditionally immortalised cortical hNPC line, CTX0E16, results predominately in the generation of glutamatergic neurons. We demonstrate that differentiated CTX0E16 neurons retain a cortical identity, supported by their gene profile and expression of a range of glutamatergic and cortical markers. In addition, differentiated CTX0E16 cells form polarised neurons, and exhibit a cellular morphology typical of pyramidal neurons previously observed in vitro [17, 26, 27]. Furthermore, our data indicate that differentiated CTX0E16 neurons express a range of neurotransmitter receptor and signalling proteins consistent with the generation of young maturing neurons. Functional mobilisation of Ca^2+^ transients and activation of ERK1/2 signalling pathways were observed in response to neurotransmitter receptor activation, indicating that CTX0E16 cells generated functional neurons. An important question to consider is whether the subsequently generated neurons closely resemble those found in vivo and if they develop functional neuronal characteristics including action potentials and synaptic connections. We have addressed these questions using several different strategies. Indeed, differentiated CTX0E16 neurons displayed the hallmarks of putative synapse formation and neuronal activity, as demonstrated by spontaneous Ca^2+^ transients, electrophysiological recordings of action potentials and EPSCs and the regulation of neuronal morphology in response to physiological stimuli. Collectively, these data demonstrate that CTX0E16 neurons are an ideal platform from which to investigate both basic and pathological mechanisms associated with neurodevelopmental disorders.

Many neurodevelopmental disorders display deficits in cortical neurons. In particular, morphological and functional properties of excitatory (glutamatergic) projection neurons in the cortex have been strongly implicated in disorders such as autism spectrum disorders (ASDs) and schizophrenia [56]. While animal models have advanced our understanding of the mechanisms that underlie these disorders, many aspects of these diseases remain unknown. Moreover, current rates of conversion of pre-clinical investigation and drug discovery have in the most part failed to deliver [57]. One approach that has been suggested to overcome such shortcomings has been to use human neurons. Indeed, several cellular models to address this have been put forward, including ESCs, iPSCs and immortalised NSCs and NPCs [1–5, 21]. Difficulties in obtaining tissue for ESCs in many cases hinder the use of these cells as a cellular model. Similarly, the generation of appropriate control and patient iPSCs and differentiation of these cells into distinct neuronal lineages is both time-consuming and costly. On the other hand, immortalised hNSC and hNPCs represent a potentially unlimited source of cells that can generate defined neural cell types [2–5]. However, in order for us to realise the utility of such cells it is critical for us to fully characterise the lineage and basic characteristics of these cells lines. Already several groups have used hNPCs to investigate the complex mechanisms underlying neurodevelopmental disorders. These include studies on cortisol and stress neurons derived from hippocampal neurons [18], DISC1 loss of function in a cortical hNPC model with implication for neuropsychiatric diseases [58], and knockdown of the schizophrenia and bipolar susceptibility gene, ZNF804a in a cortical hNPC line [20]. Based on our immunohistochemical and functional characterisation of differentiated CTX0E16 neurons, we feel that this hNPC line represents an ideal platform from which to investigate the development of human cortical neurons, and how such mechanisms may be perturbed during the early stages of pathogenesis of a range of neurodevelopmental disorders. Moreover, these cells represent an alternative cell model that has the potential to be scaled up for use in medium- and high-throughput screens.

## Additional file

Additional file 1: Figure S1. Expression profile of undifferentiated and differentiated (DD28) CTX0E16 cells. Undifferentiated CTX0E16 cells express a range of neurotransmitter receptors, signalling and disease-associated proteins, which are upregulated during differentiation, as determined by RT-PCR; n = 3 independent experiments carried out in triplicate. **Table S1** List of antibodies used in study. **Table S2** List and description of primers used in RT-PCR and q-PCR experiments. (PDF 181 kb)

## Abbreviations

4-OHT: 4-hydroxytamoxifen
AMPA: α-amino-3-hydroxy-5-methyl-4-isoxazolepropionic acid
ANOVA: analysis of variance
DAPI: 4′,6-diamidino-2-phenylindole
DD: differentiation day
DMEM: Dulbecco’s modified Eagle’s medium
EGF: epidermal growth factor
eGFP: enhanced green fluorescent protein
EPSC: excitatory post synaptic currents
FGF: fibroblast growth factor
GFP: green fluorescent protein
GPCR: G-protein-coupled receptor
hNPC: human neural progenitor cell
hNSC: human neural stem cell
NDM: neural differentiation medium
NMDA: N-methyl-D-aspartate
PDL: Poly-D-lysine
q-PCR: quantitative polymerase chain reaction
RMM: reduced modified medium
ROI: region(s) of interest
RT-PCR: reverse transcriptase polymerase chain reaction
TTX: tetrodotoxin

## References

[1] Bray NJ, Kapur S, Price J (2012). Investigating schizophrenia in a “dish”: possibilities, potential and limitations. World Psychiatry..

[2] Breier JM, Gassmann K, Kayser R, Stegeman H, De Groot D, Fritsche E (2010). Neural progenitor cells as models for high-throughput screens of developmental neurotoxicity: state of the science. Neurotoxicol Teratol..

[3] Gaspard N, Vanderhaeghen P (2011). From stem cells to neural networks: recent advances and perspectives for neurodevelopmental disorders. Dev Med Child Neurol..

[4] Ladran I, Tran N, Topol A, Brennand KJ (2013). Neural stem and progenitor cells in health and disease. Wiley Interdiscip Rev Syst Biol Med..

[5] De Filippis L, Binda E (2012). Concise review: self-renewal in the central nervous system: neural stem cells from embryo to adult. Stem Cells Transl Med..

[6] Sah DW, Ray J, Gage FH (1997). Bipotent progenitor cell lines from the human CNS. Nat Biotechnol..

[7] Carpenter MK, Cui X, Hu ZY, Jackson J, Sherman S, Seiger A (1999). In vitro expansion of a multipotent population of human neural progenitor cells. Exp Neurol..

[8] Johansson CB, Svensson M, Wallstedt L, Janson AM, Frisen J (1999). Neural stem cells in the adult human brain. Exp Cell Res..

[9] Nunes MC, Roy NS, Keyoung HM, Goodman RR, McKhann G, Jiang L (2003). Identification and isolation of multipotential neural progenitor cells from the subcortical white matter of the adult human brain. Nat Med..

[10] Vescovi AL, Parati EA, Gritti A, Poulin P, Ferrario M, Wanke E (1999). Isolation and cloning of multipotential stem cells from the embryonic human CNS and establishment of transplantable human neural stem cell lines by epigenetic stimulation. Exp Neurol..

[11] Cacci E, Villa A, Parmar M, Cavallaro M, Mandahl N, Lindvall O (2007). Generation of human cortical neurons from a new immortal fetal neural stem cell line. Exp Cell Res..

[12] Cocks G, Romanyuk N, Amemori T, Jendelova P, Forostyak O, Jeffries AR (2013). Conditionally immortalized stem cell lines from human spinal cord retain regional identity and generate functional V2a interneurons and motorneurons. Stem Cell Res Ther..

[13] De Filippis L, Lamorte G, Snyder EY, Malgaroli A, Vescovi AL (2007). A novel, immortal, and multipotent human neural stem cell line generating functional neurons and oligodendrocytes. Stem Cells..

[14] Pollock K, Stroemer P, Patel S, Stevanato L, Hope A, Miljan E (2006). A conditionally immortal clonal stem cell line from human cortical neuroepithelium for the treatment of ischemic stroke. Exp Neurol..

[15] Zhang H, Wang Y, Zhao Y, Yin Y, Xu Q (2008). Immortalized human neural progenitor cells from the ventral telencephalon with the potential to differentiate into GABAergic neurons. J Neurosci Res..

[16] Donato R, Miljan EA, Hines SJ, Aouabdi S, Pollock K, Patel S (2007). Differential development of neuronal physiological responsiveness in two human neural stem cell lines. BMC Neurosci..

[17] Gaspard N, Bouschet T, Hourez R, Dimidschstein J, Naeije G, van den Ameele J (2008). An intrinsic mechanism of corticogenesis from embryonic stem cells. Nature..

[18] Anacker C, Zunszain PA, Cattaneo A, Carvalho LA, Garabedian MJ, Thuret S (2011). Antidepressants increase human hippocampal neurogenesis by activating the glucocorticoid receptor. Mol Psychiatry..

[19] Stevanato L, Corteling RL, Stroemer P, Hope A, Heward J, Miljan EA (2009). c-MycERTAM transgene silencing in a genetically modified human neural stem cell line implanted into MCAo rodent brain. BMC Neurosci.

[20] Hill MJ, Jeffries AR, Dobson RJ, Price J, Bray NJ (2012). Knockdown of the psychosis susceptibility gene ZNF804A alters expression of genes involved in cell adhesion. Hum Mol Genet..

[21] Sinden JD, Vishnubhatla I, Muir KW (2012). Prospects for stem cell-derived therapy in stroke. Prog Brain Res..

[22] Srivastava DP, Woolfrey KM, Penzes P (2011). Analysis of dendritic spine morphology in cultured CNS neurons. J Vis Exp..

[23] ImageJ homepage. http://imagej.nih.gov/ij/

[24] UCSC Genome Bioinformatics homepage. http://genome.ucsc.edu

[25] Albizu L, Cottet M, Kralikova M, Stoev S, Seyer R, Brabet I (2010). Time-resolved FRET between GPCR ligands reveals oligomers in native tissues. Nat Chem Biol..

[26] Espuny-Camacho I, Michelsen KA, Gall D, Linaro D, Hasche A, Bonnefont J (2013). Pyramidal neurons derived from human pluripotent stem cells integrate efficiently into mouse brain circuits in vivo. Neuron..

[27] Shi Y, Kirwan P, Smith J, Robinson HP, Livesey FJ (2012). Human cerebral cortex development from pluripotent stem cells to functional excitatory synapses. Nat Neurosci..

[28] Jones EG (2009). The origins of cortical interneurons: mouse versus monkey and human. Cereb Cortex..

[29] Radonjic NV, Ortega JA, Memi F, Dionne K, Jakovcevski I, Zecevic N (2014). The complexity of the calretinin-expressing progenitors in the human cerebral cortex. Front Neuroanat..

[30] Illes S, Theiss S, Hartung HP, Siebler M, Dihne M (2009). Niche-dependent development of functional neuronal networks from embryonic stem cell-derived neural populations. BMC Neurosci..

[31] Real MA, Davila JC, Guirado S (2006). Immunohistochemical localization of the vesicular glutamate transporter VGLUT2 in the developing and adult mouse claustrum. J Chem Neuroanat..

[32] Ben-Ari Y (2002). Excitatory actions of gaba during development: the nature of the nurture. Nat Rev Neurosci..

[33] Dotti CG, Sullivan CA, Banker GA (1988). The establishment of polarity by hippocampal neurons in culture. J Neurosci..

[34] Markram H, Toledo-Rodriguez M, Wang Y, Gupta A, Silberberg G, Wu C (2004). Interneurons of the neocortical inhibitory system. Nat Rev Neurosci..

[35] Romand S, Wang Y, Toledo-Rodriguez M, Markram H (2011). Morphological development of thick-tufted layer v pyramidal cells in the rat somatosensory cortex. Front Neuroanat..

[36] Srivastava DP, Woolfrey KM, Jones KA, Anderson CT, Smith KR, Russell TA (2012). An autism-associated variant of Epac2 reveals a role for Ras/Epac2 signaling in controlling basal dendrite maintenance in mice. PLoS Biol..

[37] Threadgill R, Bobb K, Ghosh A (1997). Regulation of dendritic growth and remodeling by Rho, Rac, and Cdc42. Neuron..

[38] Horton AC, Racz B, Monson EE, Lin AL, Weinberg RJ, Ehlers MD (2005). Polarized secretory trafficking directs cargo for asymmetric dendrite growth and morphogenesis. Neuron..

[39] Horton AC, Yi JJ, Ehlers MD (2006). Cell type-specific dendritic polarity in the absence of spatially organized external cues. Brain Cell Biol..

[40] Jones KA, Eng AG, Raval P, Srivastava DP, Penzes P (2014). Scaffold protein X11-alpha interacts with kalirin-7 in dendrites and recruits it to Golgi outposts. J Biol Chem..

[41] Conn PJ, Pin JP (1997). Pharmacology and functions of metabotropic glutamate receptors. Annu Rev Pharmacol Toxicol..

[42] Beaulieu JM, Gainetdinov RR (2011). The physiology, signaling, and pharmacology of dopamine receptors. Pharmacol Rev..

[43] Andreae LC, Burrone J (2014). The role of neuronal activity and transmitter release on synapse formation. Curr Opin Neurobiol..

[44] Rosenberg SS, Spitzer NC (2011). Calcium signaling in neuronal development. Cold Spring Harb Perspect Biol..

[45] Verpelli C, Carlessi L, Bechi G, Fusar Poli E, Orellana D, Heise C (2013). Comparative neuronal differentiation of self-renewing neural progenitor cell lines obtained from human induced pluripotent stem cells. Front Cell Neurosci..

[46] Thomas GM, Huganir RL (2004). MAPK cascade signalling and synaptic plasticity. Nat Rev Neurosci..

[47] Tada T, Sheng M (2006). Molecular mechanisms of dendritic spine morphogenesis. Curr Opin Neurobiol..

[48] McAllister AK (2007). Dynamic aspects of CNS synapse formation. Annu Rev Neurosci..

[49] Yoshihara Y, De Roo M, Muller D (2009). Dendritic spine formation and stabilization. Curr Opin Neurobiol..

[50] Fattorini G, Verderio C, Melone M, Giovedi S, Benfenati F, Matteoli M (2009). VGLUT1 and VGAT are sorted to the same population of synaptic vesicles in subsets of cortical axon terminals. J Neurochem..

[51] Zander JF, Munster-Wandowski A, Brunk I, Pahner I, Gomez-Lira G, Heinemann U (2010). Synaptic and vesicular coexistence of VGLUT and VGAT in selected excitatory and inhibitory synapses. J Neurosci..

[52] Ziv NE, Smith SJ (1996). Evidence for a role of dendritic filopodia in synaptogenesis and spine formation. Neuron..

[53] Arikkath J (2009). Regulation of dendrite and spine morphogenesis and plasticity by catenins. Mol Neurobiol..

[54] Jan YN, Jan LY (2010). Branching out: mechanisms of dendritic arborization. Nat Rev Neurosci..

[55] Krey JF, Pasca SP, Shcheglovitov A, Yazawa M, Schwemberger R, Rasmusson R (2013). Timothy syndrome is associated with activity-dependent dendritic retraction in rodent and human neurons. Nat Neurosci..

[56] Penzes P, Cahill ME, Jones KA, VanLeeuwen JE, Woolfrey KM (2011). Dendritic spine pathology in neuropsychiatric disorders. Nat Neurosci..

[57] Dragunow M (2008). The adult human brain in preclinical drug development. Nat Rev Drug Discov..

[58] Kobayashi NR, Sui L, Tan PS, Lim EK, Chan J, Choolani M (2010). Modelling disrupted-in-schizophrenia 1 loss of function in human neural progenitor cells: tools for molecular studies of human neurodevelopment and neuropsychiatric disorders. Mol Psychiatry..

