# Supplementary material for: Characterisation of neurons derived from a cortical human neural stem cell line CTX0E16
Source: Stem Cell Res Ther. 2015 Aug 22;6(1):149. doi: 10.1186/s13287-015-0136-8 (PMC4546258; doi:10.1186/s13287-015-0136-8)
Supplement: Additional file 1: Figure S1. — Expression profile of undifferentiated and differentiated (DD28) CTX0E16 cells. Undifferentiated CTX0E16 cells express a range of neurotransmitter receptors, signalling and disease-associated proteins, which are upregulated during differentiation, as determined by RT-PCR; n = 3 independent experiments carried out in triplicate. Table S1 List of antibodies used in study. Table S2 List and description of primers used in RT-PCR and q-PCR experiments. (PDF 181 kb) [file 13287_2015_136_MOESM1_ESM.pdf]

Characterisation of neurons derived from a cortical human neural stem cell line CTX0E16

Greg W. Anderson[1]*, P. J. Michael Deans[1]*, Ruth D. T. Taylor[2], Ding Chen[1], Pooja Raval[1], Harrison Lowder[1], Srishti Murkerji[1], Laura C. Andreae[2], Deepak. P. Srivastava[1]§, Brenda P. Williams[1]§.

[1]Department of Basic and Clinical Neuroscience, Institute of Psychiatry Psychology and Neuroscience, and [2]MRC Centre for Developmental Neurobiology, King's College London, London, SE5 8AF, UK

* = equal contribution

§ Address correspondence to: Deepak P Srivastava, 125 Coldharbour Lane, London, SE5 9NU, deepak.srivsatava@kcl.ac.uk; or Brenda P. Williams 125 Coldharbour Lane, London, SE5 9NU, brenda.p.williams@kcl.ac.uk

Supplemental Figure 1. Expression profile of undifferentiated and differentiated (DD28) CTX0E16 cells. Undifferentiated CTX0E16 cells express a range of neurotransmitter receptors, signalling and disease associated proteins, which are upregulated during differentiation, as determined by RT-PCR. N = 3 independent experiments carried out in triplicate.

A

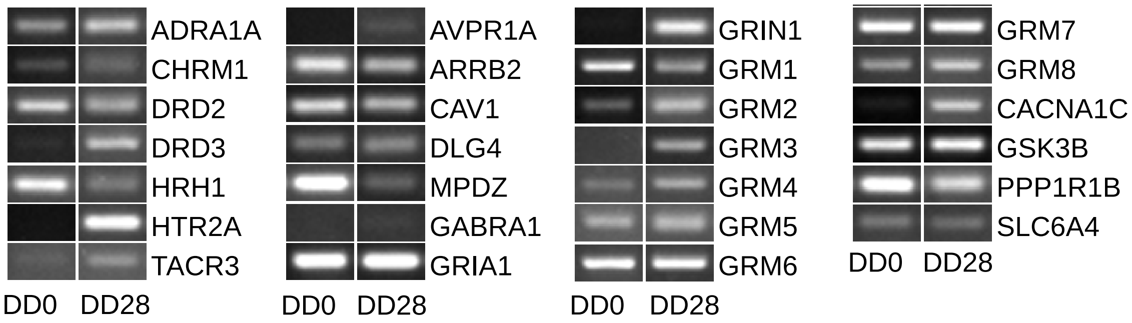

Anderson, Deans et al. Supplemental Figure 1

Supplemental Table 1. List of Antibodies used in study.

Antibody	Company	Catalogue No.	Dilution
chicken α GFP	Abcam	ab13970	1:10000
chicken α MAP2	Abcam	AB104896	1:1000
mouse α Bassoon	Assay Design	SAP7F407	1:300
mouse α CaMKII	Abcam	ab52476	1:500
mouse α GluA2	NeuroMab/Antibody Inc	L21/32	1:500
mouse α GluN1	NeuroMab/Antibody Inc	N308/48	1:500
mouse α GM130	BD Biosciences	610823	1:250
mouse α Nestin	Millipore	MAB5326	1:500
mouse α O4	Sigma	O7139	1:1000
mouse α PSD-95	NeuroMab/Antibody Inc	K28/43	1:1000
mouse α SAP97	NeuroMab/Antibody Inc	K64/15	1:250
mouse α SNAP-25	Synaptic-Systems	111 011	1:1000
mouse α VAMP2	Synaptic-Systems	104 211	1:2000
mouse α VGlut1	Millipore	MAB5502	1:250
mouse α βIII Tubulin	Millipore	MAB1637	1:1000
rabbit α Calbindin D-28k	SWant	CB-38a	1:20000
rabbit α Calretinin	SWant	7699/4	1:10000
rabbit α Cux1	Santa Cruz	sc-13024	1:500
rabbit α Doublecortin	Abcam	ab18723	1:500
rabbit α GABA	Sigma	A2052	1:1000
rabbit α GAD65/67	Millipore	AB1511	1:250
rabbit α Gephyrin	Millipore	AB5725	1:500
rabbit α GluA1	Millipore	AB1504	1:500
rabbit α Glutamate	Sigma	G6642	1:500
rabbit α KI-67	Abcam	ab15580	1:500
rabbit α PSD-95	Cell Signalling	25075	1:1000
rabbit α S100β	Dako	M7221	1:500
rabbit α SOX2	Millipore	AB5603	1:100
rabbit α Synapsin-1	Cell Signalling	5297	1:200
rabbit α Tau	Dako	A0024	1:500
rabbit α Tbr1	Abcam	ab31940	1:200
rabbit α VGAT	Millipore	AB5062P	1:300
rabbit α VGlut2	Abcam	ab101756	1:500
rat α CTIP2	Abcam	ab28448	1:500

Supplemental Table 2. List and description of Primers used in RT- and q-PCR experiments.

Gene	Protein	Sequence (5' – 3')
ADRA1A	Adrenergic α_{1A} receptor (GPCR): high-affinity target for first and second generation antipsychotic drugs and may contribute to efficacy and side effect liability	GAGAAGAAAGCGG CCAAAAC AGGGCTTGAAATCA GGGAAG
ARRB2	β-Arrestin 2 (scaffold and signalling protein): multifunctional scaffolding protein involved in GPCR desensitisation and G-protein independent signalling	TGTGGCTCAACTCG AACAAG CTGAGCAGTGGGG TTATGGT
AVPR1A	Arginine vasopressin receptor V_{1A} (GPCR): Current interest as a target for the treatment of autism and schizophreniaReceptor target of novel compounds to treat autism	TTTGTGATCGTGAC GGCTTA TGGTGATGGTAGG GTTTTCC
CACNA1C	L-type, voltage-dependent calcium channel α_{1C} subunit: One of the major voltage-dependent Ca^{2+} channel found in the CNS. Recently associated with risk of developing schizophrenia and bipolar disorder	CCTGAGAATGAGGA CGAAGG GTTTTCGGTGTTGA CGGACT
CAMK2A	Calcium/Calmodulin-Dependent Protein Kinase II α is a serine/threonine protein kinase crucial for several aspects of plasticity at glutamatergic synapses	GAGCCATTCTCACC ACGATGCT TGGTGTTGGTGCTC TCTGAGGA
CAV1	Caveolin-1 (scaffold protein): Accessory protein involved in GPCR plasma membrane expression and internalisation	GCGACCCTAAACAC CTCAAC CAAATGCCGTCAAA ACTGTG
CHRM1	Cholinergic muscarinic M_1 receptor (GPCR): mixed-affinity target for range of antipsychotics. Selective compounds under investigated to treat cognitive symptoms and as possible monotherapy	GGACCCTACAGACC CCTCTT GTGTTCATGGTGGC TAGGTG
DLG4	Postsynaptic density protein 95 (PSD-95): Scaffold protein important for GPCR localisation and function	TCTCCCACACACAT TCCAGA ACCCTGCCTTAGGA GAGAGC
DRD2	Dopamine D_2 receptor (GPCR): high-affinity target for first and second generation antipsychotic drugs and associated with clinical efficacy	ATCTCCTGCCCACT CCTCTT TGACAATGAAGGGC ACGTAG
DRD3	Dopamine D_3 receptor (GPCR): high-affinity target for first and second generation antipsychotic drugs, difficult to functionally distinguish from dopamine D_2 receptors	TGCCTATGCCTACT CCATCC GAGTGCTGCTTTTC CAGCTT
EMX1	Homeobox protein EMX1 is a is a transcription factor expressed in the telencephalon. Emx1 plays a role in specification of positional identity, the proliferation of neural stem cells and differentiation of layer-specific neuronal phenotypes, especially those found in layers V and VI	GCCTTCGAGAAGAA CCACTACG CGGTTCTGGAACCA CACCTTCA
GABRA1	GABA receptor subunit α_1 (ionotropic): Ubiquitous	CCATGAGGCTGACA GTGAGA

	subunit for all GABA-A receptors – contains binding site for benzodiazepine anxiolytics	ATTTTAGTGGGCAA GCATGG
GRIA2	Glutamate receptor 2 (AMPA receptor subunit, ionotropic): allosteric modulators of this receptor currently under investigation as possible adjunct treatment for cognitive deficits and monotherapy	TTCAGATGAGACCC GACCTC GCACAGCTTGCAGT GTTGAT
GRIN1	Glutamate receptor, N-methyl D-aspartate (NMDA) 1 (NR1, ionotropic): allosteric modulators currently in phase II clinical trial for the treatment of cognitive and negative symptoms	AGATGGCTCTGTCG GTGTG GTGGGAGTGAAGT GGTCGTT
GRM1	Metabotropic glutamate receptors 1 (mGluR1) Metabotropic glutamate receptors 1-8 ($mGluR_{1-8}$, GPCRs): several of these are targeted by compounds currently in clinical and preclinical development for the treatment of schizophrenia – particularly $mGluR_2$, $mGluR_3$ and $mGluR_5$	CTAGCTGGCATCTT CCTTGG<br><br>TGAGGCAATGATCA CCTGAG
GRM2	Metabotropic glutamate receptors 2 (mGluR2)	CCGCAGAAGAACGT GGTTAG GCAAACAGTGGGG ACAAACT
GRM3	Metabotropic glutamate receptors 3 (mGluR3)	GGTCTGATCCTGGT GCAAAT TTTGAAGGCGTACA CAGTGC
GRM4	Metabotropic glutamate receptors 4 (mGluR4)	TCTGTCTCCAGCCC TGTCTT ACAGGAAAGAAAAC GGCAGA
GRM5	Metabotropic glutamate receptors 5 (mGluR5)	ATCAGCGAGCTCAA CTCCAT CTGGATCTCTTTGG GGATCA
GRM6	Metabotropic glutamate receptors 6 (mGluR6)	ATTGGGCAGTGTGG AAGAAC CCCAGTTCCTCAGC TCACTC
GRM7	Metabotropic glutamate receptors 7 (mGluR7)	CGGCGCTATGACTT CTTCTC CTGCCTCTTTGGAA ATCTGC
GRM8	Metabotropic glutamate receptors 8 (mGluR8)	GAGGATATTGGAAG CAGCAAA TCAATTGATGCTCG TTTGG
GSK3B	Glycogen synthase kinase 3β (GSK-3β): Important signalling node for neuronal activity and several neuropsychiatric drugs. Aberrant function has been linked to the schizophrenia	GAGACCGTGGACA GACCAAT ACTGGTGGTTTTTC CTGTGC
HRH1	Histamine H_1 receptor (GPCR): high-affinity target for first and second generation antipsychotic drugs and associated with weight gain and metabolic side effects	CACACTGAACCCCC TCATCT ATTTTGTTGCATCC CCTCAG

HTR2A	5-HT_{2A} receptor (GPCR): high-affinity target for second and some first generation antipsychotic drugs, and has been associated with atypicality	CAGAATCCCATCCA CCACA<br>TCCTGTAGCCCAAA GACTGG
MPDZ	Multiple PDZ domain protein (MUPP1): Scaffold protein important for GPCR localisation and function	CCATAGTTTCCCGT TCTCCA<br>CAATCGTTTCCATG TGTTGC
PPP1R1B	Protein phosphatase 1 regulatory subunit 1B (PPP1R1B), aka. dopamine- and cAMP-regulated neuronal phosphoprotein (DARPP-32)	CCCTAAGATCTGGG GAAAGG<br>CTGGTAGAAGCCG GTGAGAG
SLC6A4	5-HT transporter (SERT): Primary target of selective serotonin reuptake inhibitor (SSRI) antidepressants	TGCCACCTTCCTGT CTCTCT<br>GCCAAGCCATGGT GACTAAT
TACR3	Tachykinin 3 receptor (NK_3, GPCR): target of compounds currently in phase II clinical trial for the treatment of schizophrenia	AGGCCGTACTCTCT GCTTTG<br>TGATGAGCAATGGG AAACAG
TBR1	T-box, brain, 1 is a transcription factor important in vertebrate embryo development. This gene is also known by several other names: T-Brain 1, TBR-1, TES-56, and MGC141978. TBR1 interacts with various genes and proteins in order to regulate cortical development, specifically within layer VI of the developing six-layered human cortex.	TAACAATGGGCAGA TGGTGG<br>AGGGAAAGTGAAC GTCTGCA
GAPDH	Used as housekeeping gene in q-PCR	CCTGACCTGCCGTC TAGAAA<br>ACCTGGTGCTCAGT GTAGCC
HPRT1	Used as housekeeping gene in q-PCR	TGACACTGGCAAAA CAATGCA<br>GGTCCTTTTCACCA GCAAGCT
RPL13A	Used as housekeeping gene in q-PCR	CCTGGAGGAGAAG AGGAAAGAGA<br>TTGAGGACCTCTGT GTATTTGTCAA

List of forward (F) and reverse (R) primer sequences used in gene expression studies; forward primer is described first. A brief description of the encoding protein and potential relevance for disease biology is also given.
